# Wnt5a induces ROR1 to recruit cortactin to promote breast-cancer migration and metastasis

**DOI:** 10.1038/s41523-019-0131-9

**Published:** 2019-10-25

**Authors:** Md Kamrul Hasan, George F. Widhopf, Suping Zhang, Sharon M. Lam, Zhouxin Shen, Steven P. Briggs, Barbara A. Parker, Thomas J. Kipps

**Affiliations:** 1 Moores Cancer Center, University of California San Diego, La Jolla, CA USA; 2 Guangdong Key Laboratory for Genome Stability & Disease Prevention, Department of Pharmacology, International Cancer Center, Shenzhen University Health Science Center, Shenzhen, 518060 Guangdong China; 3 Section of Cell and Developmental Biology, University of California San Diego, La Jolla, CA USA

**Keywords:** Breast cancer, Metastasis, RHO signalling

## Abstract

ROR1 is a conserved oncoembryonic surface protein expressed in breast cancer. Here we report that ROR1 associates with cortactin in primary breast-cancer cells or in MCF7 transfected to express ROR1. Wnt5a also induced ROR1-dependent tyrosine phosphorylation of cortactin (Y421), which recruited ARHGEF1 to activate RhoA and promote breast-cancer-cell migration; such effects could be inhibited by cirmtuzumab, a humanized mAb specific for ROR1. Furthermore, treatment of mice bearing breast-cancer xenograft with cirmtuzumab inhibited cortactin phosphorylation in vivo and impaired metastatic development. We established that the proline at 841 of ROR1 was required for it to recruit cortactin and ARHGEF1, activate RhoA, and enhance breast-cancer-cell migration in vitro or development of metastases in vivo. Collectively, these studies demonstrate that the interaction of ROR1 with cortactin plays an important role in breast-cancer-cell migration and metastasis.

## Introduction

ROR1 is an evolutionarily conserved, developmentally restricted type-I receptor–tyrosine-kinase-like orphan receptor,^1–4^ which has a cytoplasmic domain consisting of a tyrosine-kinase-like domain, two serine/threonine-rich domains, and a proline-rich domain (PRD). With few exceptions,^5^ ROR1 expression is the highest during embryogenesis, diminishes during fetal development, and becomes negligible on most postpartum tissues.^6^

We found that ROR1 is expressed by most human cancers, including breast cancer, intimating that it plays a role in cancer pathophysiology.^7^ In support of this proposition are findings demonstrating that expression of ROR1 can enhance epithelial–mesenchymal transition and cancer-cell proliferation, migration, and metastasis.^8,9^ Moreover, high-level tumor-cell expression of ROR1 associates with adverse outcome in patients with various cancers.^8,10–12^

We discovered that Wnt5a can act as a ligand for ROR1,^6^ which could induce activation of RhoA and enhance migration of breast-cancer-cell lines.^13,14^ Recently, we described that Wnt5a could cause ROR1 to associate with cortactin to enhance migration of chronic lymphocytic leukemia (CLL) cells.^15^ In this case, cortactin cooperates with another cytoskeletal protein named hematopoietic cell-specific protein-1 (HS1) to complex with ROR1 at proline 841, and thereby enhances activation of RhoA and the migration of CLL cells.^15,16^ However, HS1 is expressed primarily by hematopoietic cells, but not breast-cancer cells.^17^ As such, it is not known whether the interaction of ROR1 with cortactin can play a role in the migration or metastasis of cancer cells independent of HS1.

Cortactin (also known as EMS1, or CTTN) is expressed by the neoplastic cells of a variety of different cancers and evidently functions in cancer-cell migration and metastasis.^18–22^ Upon external stimulation, cortactin undergoes tyrosine phosphorylation and then contributes to the ordered rearrangement and polymerization of the actin cytoskeleton, which is required for cell migration.^23–27^ Cortactin contains a Src-homology-3 (SH3) domain, allowing it to bind at characteristic –P–X–X–P– motifs in the PRD of surface-receptor proteins.^28–30^ Cortactin is also expressed in breast-cancer and in breast-cancer-cell lines, such as MCF7. Amplification of the gene encoding cortactin is observed in at least 15% of metastatic breast carcinomas.^31,32^ High-level breast-cancer-cell expression and phosphorylation of cortactin associates with an unfavorable prognosis for patients with breast cancer.^33–36^ In this study, we examined whether Wnt5a could stimulate ROR1 to complex with cortactin and thereby recruit and activate ARHGEF1 to enhance activation of RhoA and promote breast-cancer-cell migration/metastasis.

## Results

### Wnt5a induces ROR1 to complex with cortactin in breast-cancer PDX

We examined eight primary breast-cancer patient-derived xenografts (PDXs) (Supplementary Fig. 1). Table 1 provides the diagnosis, histologic grade, patient’s age and race, and tumor-cell expression of estrogen receptors (ER), progesterone receptors (PR), HER2, ROR1, cortactin, or Wnt5a (see also Supplementary Fig. 1). Only one PDX (PDX1) was HER2^+^, one (PDX5) was ER^+^, and six (PDX3–8) expressed high-level ROR1. All PDX were histologic grade 3 and expressed cortactin and Wnt5a. None of the PDX were PR^+^.

**Table 1:** Data on breast-cancer patient-derived xenograft (PDX), including tumor diagnosis, grade, sex, age, race, and expression information of ER (estrogen receptor), PR (progesterone receptor), HER2, ROR1, cortactin, or Wnt5a. Here, “yes” means “expressed”, and “no” means “not expressed”

PDX samples	Diagnosis	Grade	Sex	Age	Race	ER	PR	HER2	ROR1	Cortactin	Wnt5a
1	Invasive ductal carcinoma	Grade 3	Female	73	White	No	No	Yes	No	Yes	Yes
2	Invasive ductal carcinoma	Grade 3	Female	64	White	No	No	No	No	Yes	Yes
3	Mixed ductal carcinoma	Grade 3	Female	52	White	No	No	No	Yes	Yes	Yes
4	Invasive ductal carcinoma	Grade 3	Female	44	Asian or Pacific Islander	No	No	No	Yes	Yes	Yes
5	Metastatic	Grade 3	Female	48	White	Yes	No	No	Yes	Yes	Yes
6	Invasive ductal carcinoma	Grade 3	Female	70	White	No	No	No	Yes	Yes	Yes
7	Invasive ductal carcinoma	Grade 3	Female	68	Asian or Pacific Islander	No	No	No	Yes	Yes	Yes
8	Invasive ductal carcinoma	Grade 3	Female	32	White	No	No	No	Yes	Yes	Yes

Characteristics of breast-cancer patient-derived xenograft (PDX) samples

**Fig. 1:**
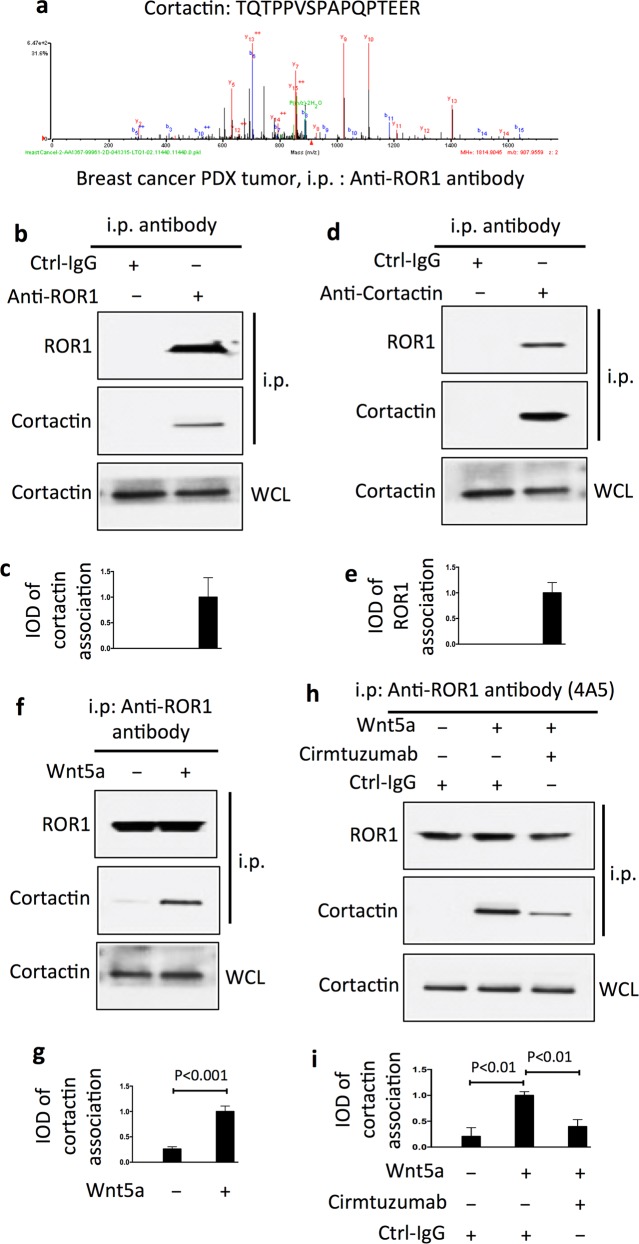
Association of ROR1 with cortactin in breast-cancer patient-derived xenografts (PDXs). **a** Cortactin peptide identified by 2D-nanoLC–MS/MS in anti-ROR1 (cirmtuzumab) immune precipitates (i.p.) of lysates of PDX4 (representative of two PDXs). **b** Immunoblot of i.p. by using anti-ROR1 mAb or control IgG (Ctrl-IgG), as indicated on the top, by using lysates of PDX3 (representative of three PDXs). The bottom panel is an immunoblot of the whole-cell lysates (WCL) probed with anti-cortactin mAb. **c** Black columns indicate the mean relative interaction of cortactin with ROR1 (error bars indicate S.D.) for PDX3, PDX4, and PDX5. **d** Immunoblot of i.p. by using anti-ROR1 mAb or Ctrl-IgG, as indicated on the top, by using lysates of PDX4 (representative of three PDXs). The bottom panel is an immunoblot of the WCL probed with anti-cortactin mAb. **e** Black columns indicate the mean relative interaction of cortactin with ROR1 (±S.D.) for PDX3, PDX4, and PDX5. **f** Immunoblot of anti-ROR1 i.p. from lysates of serum-starved PDX5 cells (representative of three PDXs) that subsequently were treated for 30 min without (−) or with (+) Wnt5a (100 ng/ml), as indicated on the top. The bottom panel is an immunoblot of the WCL probed with anti-cortactin mAb. **g** Columns indicate the mean relative interaction of cortactin with ROR1 (±S.D.) for PDX3, PDX4, and PDX5 that were treated for 30 min without (−) or with (+) Wnt5a, as in 1f and indicated below (*P* < 0.001, two-tailed Student’s *t* test). **h** Immunoblot of anti-ROR1 (4A5) i.p. by using lysates of serum-starved PDX5 (representative of three PDXs) that had been treated with Ctrl-IgG or cirmtuzumab (10 μg/ml) for 2 h, and subsequently treated for 30 min without (−) or with (+) Wnt5a (100 ng/ml), as indicated above. **i** Columns indicate the mean relative interaction of cortactin with ROR1 (±S.D.) for PDX3, PDX4, and PDX5 that had been treated with Ctrl-IgG or cirmtuzumab, without (−) or with (+) Wnt5a, as in 1 h, and indicated below (*P* < 0.01, two-tailed Student’s *t* test)

Mass spectrometry (MS) analyses of anti-ROR1 mAb immune precipitates (i.p.) from lysates of ROR1-expressing breast-cancer PDX revealed cortactin in addition to ROR1 (Fig. 1a). Immunoblot analyses of anti-ROR1 or anti-cortactin immune precipitates confirmed that cortactin complexed with ROR1 in breast-cancer PDX cells (Fig. 1b–e). However, we did not detect such ROR1–cortactin complexes in lysates prepared from breast-cancer–PDX cells cultured overnight in serum-free media unless they were treated with exogenous Wnt5a (Fig. 1f, g), suggesting that the endogenous Wnt5a was limiting and insufficient to maintain ROR1–cortactin complexes in vitro.

We treated the breast-cancer PDX cells with cirmtuzumab, a humanized mAb specific for a functional epitope of ROR1 that is distinct from the epitope recognized by the anti-ROR1-mAb 4A5, which we used to generate the anti-ROR1 i.p. We found that treatment of the breast-cancer PDX with cirmtuzumab blocked Wnt5a from inducing ROR1 to complex with cortactin (Fig. 1h, i).

### Wnt5a stimulates ROR1-dependent cortactin phosphorylation and enhances the migration of breast-cancer PDX cells

**Fig. 2:**
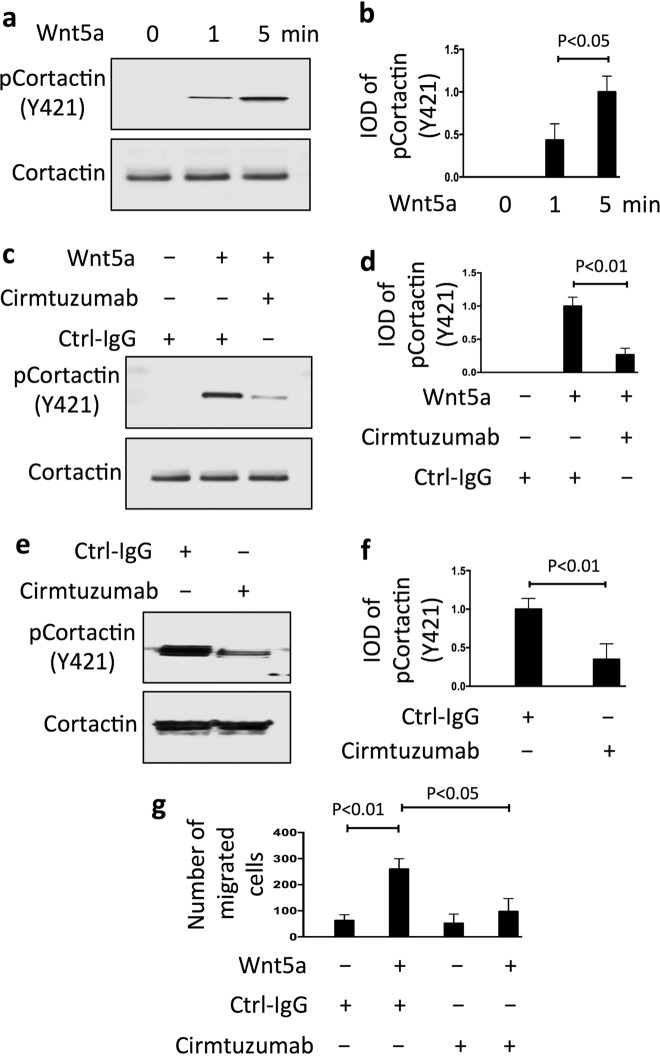
Wnt5a induces ROR1-dependent phosphorylation of cortactin and enhances migration of breast-cancer PDX cells. **a** Immunoblot analysis of lysates prepared from serum-starved PDX5 (representative of three PDXs) that subsequently were treated with Wnt5a (100 ng/ml) for the times indicated above (in minutes). **b** Columns indicate the mean relative tyrosine phosphorylation of cortactin at Y421 (pCortactin) (±S.D.) for PDX4, PDX5, and PDX6 treated for 0, 1, or 5 min with Wnt5a (*P* < 0.05, two-tailed Student’s *t* test). **c** Immunoblot analysis of lysates prepared from serum-starved PDX5 (representative of three PDXs) that subsequently were treated with Ctrl-IgG or cirmtuzumab (10 μg/ml), without (−) or with (+) Wnt5a, as indicated above. **d** Columns indicate the mean relative pCortactin (±S.D.) for PDX4, PDX5, and PDX6 cells treated with Ctrl-IgG or cirmtuzumab for 2 h, and subsequently treated for 5 min without (−) or with (+) Wnt5a, as indicated below (*P* < 0.01, Student’s *t* test). **e** Immunoblot analysis of lysates of PDX4 harvested from mice treated with Ctrl-IgG or cirmtuzumab (10 mg/kg), as indicated on top, and probed for pCortactin or Cortactin, as indicated on the left. **f** Columns indicate the mean relative pCortactin (±S.D.) for PDX3, PDX4, and PDX5 (*P* < 0.01, two-tailed Student’s *t* test). **g** Columns indicate the mean cell migration at 10 h (±S.D.) in the absence (−) or presence (+) of exogenous Wnt5a (200 ng/ml) for serum-starved PDX4, PDX5, and PDX6 that were treated with Ctrl-IgG or cirmtuzumab (10 μg/ml). Data are from three independent experiments (*P* < 0.05; *P* < 0.01, Student’s *t* test)

Previous studies found that cortactin may be phosphorylated in breast-cancer cells of some patients,^23,31^ and that high levels of phosphorylated cortactin associated with enhanced cancer-cell migration, metastasis, and adverse prognosis.^18,20,33^ We found breast-cancer PDX that expressed high levels of ROR1 had higher levels of Y421-phosphorylated cortactin than breast-cancer PDX with low levels of ROR1 (Supplementary Fig. 1). Culture of breast-cancer PDX cells in serum-free media resulted in the samples having lower levels of Y421-phosphorylated cortactin over time (Supplementary Fig. 2a). However, treatment of serum-starved breast-cancer PDX cells with exogenous Wnt5a could enhance the level of phosphorylated cortactin within 5 min (Fig. 2a, b). Because prior studies indicated that ROR1 was a pseudokinase with activity that was dependent on its ability to associate with other kinases, such as Src,^37,38^ we examined for this by reducing expression of Src by using specific siRNA, and found that this inhibited Wnt5a-induced tyrosine phosphorylation of cortactin (Y421). These data support the notion that Src contributes to Wnt5a–ROR1-dependent cortactin phosphorylation (Supplementary Fig. 3).

Nonetheless, we found that cirmtuzumab could inhibit the capacity of Wnt5a to induce tyrosine phosphorylation of cortactin in serum-starved ROR1+breast-cancer PDX, even at Wnt5a concentrations of 100 ng/ml (Fig. 2c, d), indicating that the Wnt5a-induced phosphorylation of cortactin was dependent on ROR1. We also found that cirmtuzumab could also inhibit Y421 phosphorylation of cortactin in secondary tumors generated from primary breast-cancer PDX cells in vivo (Fig. 2e, f).

Other studies demonstrated that Wnt5a could enhance migration of breast-cancer cells.^13,14^ In this study, we found that Wnt5a enhances the migration of serum-starved breast-cancer PDX cells that expressed ROR1, and that such effects could also be blocked by cirmtuzumab (Fig. 2g).

### Wnt5a stimulates ROR1 to complex with cortactin in MCF7–ROR1 cells

MCF7 cells were generated from breast ductal carcinoma cells and have been used for studying breast-cancer-cell biology.^8,9,13^ We found that these cells do not express ROR1, but could be made to express ROR1 upon stable transfection with a ROR1-expression vector, thus generating MCF7–ROR1 (Supplementary Fig. 4).^8,9^

Immunoblot analysis of immune precipitates by using anti-ROR1 or anti-cortactin antibodies revealed that cortactin complexed with ROR1 in MCF7–ROR1 cells (Supplementary Fig. 5a, b). Such complexes were not detected in the i.p. of lysates from MCF7 cells, which lack detectable ROR1 (Supplementary Figs. 1a, 4 and 5c), or lysates from MCF7–ROR1 cells cultured overnight in serum-free media unless the cells were treated with exogenous Wnt5a (Supplementary Fig. 5d). Treatment of MCF7–ROR1 cells with cirmtuzumab blocked the capacity of Wnt5a to induce ROR1 to recruit cortactin in such serum-starved MCF7–ROR1 cells (Supplementary Fig. 5e).

### Wnt5a stimulates ROR1-dependent cortactin phosphorylation and increases the migration capacity of MCF7–ROR1 cells

We found that cortactin is phosphorylated at Y421 in MCF7–ROR1 cells cultured in DMEM with 10% fetal bovine serum (FBS). Culture of MCF7–ROR1 cells in serum-free media reduced the levels of phosphorylated cortactin over time (Supplementary Fig. 2b). Treatment of serum-starved MCF7–ROR1 cells, but not MCF7 cells, with exogenous Wnt5a induced tyrosine phosphorylation of cortactin in a time-dependent manner (Supplementary Fig. 6a). However, Wnt5a could not stimulate tyrosine phosphorylation of cortactin in MCF7–ROR1 cells treated with cirmtuzumab (Supplementary Fig. 6b), indicating that Wnt5a-induced cortactin phosphorylation was dependent on ROR1.

We found that treatment with Wnt5a also enhanced the migration of MCF7–ROR1 cells (Supplementary Fig. 7a–c). We selected the concentration of Wnt5a, 200 ng/ml, for use in our migration assays to enhance the sensitivity for detecting differences in the numbers of migrated cells over the time of the assay (Supplementary Fig. 8). However, Wnt5a had only a modest effect on the migration of MCF7, as observed in previous studies.^13^ The capacity of Wnt5a to enhance migration of MCF7–ROR1 cells specifically could be blocked by treatment with cirmtuzumab (Supplementary Fig. 6c) or cortactin-specific siRNA, but not nonspecific siRNA (Supplementary Fig. 6d, e). Collectively, these studies demonstrate that Wnt5a can enhance migration of MCF7–ROR1 cells significantly more than MCF7 cells in a ROR1/cortactin-dependent manner.

### Wnt5a stimulates ROR1/cortactin to complex with ARHGEF1 and activate RhoA

In a prior study, we described that ROR1/cortactin complex contributed to enhanced activation of RhoA in CLL cells.^15^ Here, we examined in different cellular microenvironments whether this complex could associate with ARHGEF1 to enhance activation of RhoA. Immunoblot analysis of anti-cortactin or anti-ARHGEF1 i.p. by using lysates made from ROR1^+^ breast-cancer PDX cells revealed that ARHGEF1 associated with cortactin (Supplementary Fig. 9a, b), and more specifically Y421-phosphorylated cortactin (Supplementary Fig. 9c). We noted that the ARHGEF1 i.p. generated from lysates of breast-cancer PDX treated with cortactin siRNA had less capacity to generate activated RhoA than the ARHGEF1 i.p. of PDX treated with control, nonspecific siRNA (Supplementary Fig. 9d). Moreover, Wnt5a induced less RhoA–GTP in MCF7–ROR1 cells treated with cortactin-specific siRNA than in MCF7–ROR1 cells treated with nonspecific control siRNA (si-Ctrl) (Supplementary Fig. 9e). Furthermore, Wnt5a-induced activation of RhoA could be blocked by cirmtuzumab in PDX cells (Supplementary Fig. 9f, g). We also noted that exogenous Wnt5a could induce activation of RhoA in MDA-MB-231 cells, which prior studies found expressed ROR1,^9^ but not in MDA-MB-231 cells silenced for ROR1 with ROR1-specific siRNA (Supplementary Fig. 10).

### P841 is required for ROR1 to bind and activate cortactin/ARHGEF1

**Fig. 3:**
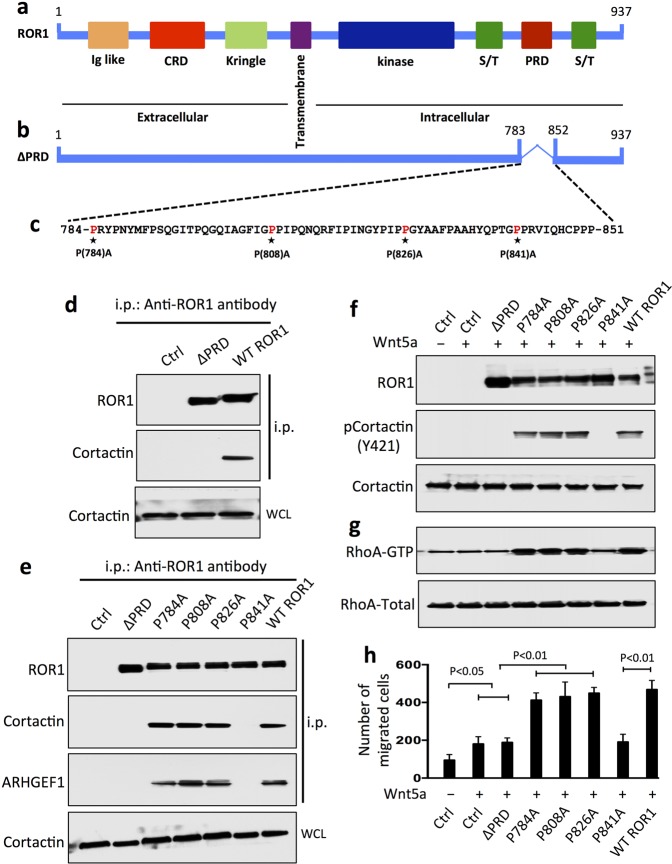
ROR1^P(841)A^ fails to associate with cortactin, induce phosphorylation of cortactin, or enhance migration of MCF7 cells. **a** Schematic depicts the structure of ROR1 protein with different domains. **b** ΔPRD represents the truncated form of ROR1 without its proline-rich region (PRD). **c** Amino acid sequences of the PRD of ROR1. Asterisks indicate the proline (P) residues that were replaced with alanine (A). **d** Immunoblots of anti-ROR1 i.p. by using lysates of MCF7 (Ctrl), MCF7–WT ROR1, or MCF7 cells transfected with ROR1–ΔPRD, as indicated on the top, and probed with antibodies specific for ROR1 or cortactin, as indicated on the left. An immunoblot of the WCL probed with anti-cortactin mAb is in the bottom panel. **e** Immunoblots of anti-ROR1 i.p. by using lysates of MCF7-Ctrl, MCF7–ΔPRD, MCF7–WT ROR1, or MCF7 transfected with each of the various mutated forms of ROR1, as indicated on the top, and probed with antibodies specific for ROR1, cortactin, or ARHGEF1, as indicated on the left. An immunoblot of the WCL probed with anti-cortactin is in the bottom panel. **f** Immunoblots of lysates prepared from serum-starved MCF7 (Ctrl), MCF7–ΔPRD, MCF7–WT ROR1, or MCF7 cells transfected with each of the various mutated forms of ROR1, as indicated on top, which subsequently were treated without (−) or with (+) Wnt5a (100 ng/ml), as indicated on the top; the membranes were probed with antibodies specific for ROR1, Cortactin, or pCortactin, as indicated on the left. **g** Immunoblot of activated RhoA (RhoA–GTP, top panel) or total RhoA (RhoA total, bottom panel) found in lysates of MCF7 (Ctrl), MCF7–ΔPRD, MCF7–WT ROR1, or MCF7 expressing any one of the various mutant forms of ROR1, as indicated on top of each lane in Fig. 1f. **h** Columns indicate the mean number of migrated cells at 10 h of MCF7 (Ctrl), MCF7–ΔPRD, MCF7 expressing any one of the various mutant forms of ROR1, or MCF7–WT ROR1, as indicated below, which were serum-starved and then examined for migration without (−) or with (+) Wnt5a (200 ng/ml), as indicated at the bottom. Data are shown from three independent experiments (*P* < 0.05; *P* < 0.01, as calculated by one-way ANOVA with Bonferroni’s multiple-comparison test).

Cortactin contains a SH3 domain, which can bind to –P–X–X–P– sites that typically are found within the PRDs of other proteins, including ROR1.^1,28–30^ Previously we described that PRD or proline at 841 of ROR1 was required for ROR1/cortactin association and phosphorylation of cortactin in CLL cells.^15^ Here, we examined whether the ROR1–PRD was necessary for ROR1 to complex with cortactin in breast-cancer cells. Accordingly, we transfected MCF7 cells with an expression vector driving expression of either wild-type ROR1 or a truncated ROR1 housing a deletion of the entire PRD (∆PRD–ROR1) (Fig. 3a, b; Supplementary Fig. 4). In contrast to the anti-ROR1 i.p. from lysates of MCF7–ROR1 cells, the anti-ROR1 i.p. from lysates of MCF7–∆PRD–ROR1 cells did not have detectable cortactin (Fig. 3d).

Accordingly, we analyzed various mutants of ROR1 that each had a substitution of alanine (A) for proline (P) at position 784, 808, 826, or 841 in one of the putative SH3-binding sites within the ROR1–PRD (Fig. 3c; Supplementary Fig. 4). Comparable amounts of ROR1 were expressed by MCF7 cells transfected to express the wild-type (W/T) or any one of the ROR1 mutants (Fig. 3e; Supplementary Fig. 4). Following treatment with Wnt5a, ROR1 with a $P =\hskip -2pt> {\text{A}}$ substitution at 784, 808, or 826 each could complex with cortactin and recruit ARHGEF1 as effectively as the W/T ROR1 (Fig. 3e). However, the mutant with a $P =\hskip -2pt> {\text{A}}$ substitution at 841 of ROR1 (ROR1^P(841)A^) did not associate with cortactin (Fig. 3e).

We examined whether Wnt5a could induce phosphorylation of cortactin or activation of RhoA in MCF7 cells transfected with W/T ROR1 or any one of the ROR1 mutants. Each of the various transfected MCF7 cell lines expressed levels of cortactin that were comparable to that of the MCF7 parental cell line (Fig. 3f). We observed that Wnt5a induced phosphorylation of cortactin and activation of RhoA, and enhanced the motility of MCF7 cells expressing ROR1 with a $P =\hskip -2pt> {\text{A}}$ substitution at 784, 808, or 826 as effectively as MCF7–ROR1 cells expressing W/T ROR1 (Fig. 3f, g, h). However, Wnt5a did not induce such effects with MCF7–∆PRD–ROR1 cells, MCF7–ROR1^P(841)A^ cells, or MCF7 cells, which lacked ROR1 altogether (Fig. 3f, g, h).

### P841 is required for ROR1 to enhance MCF7-metastatic development, which can be inhibited by cirmtuzumab

**Fig. 4:**
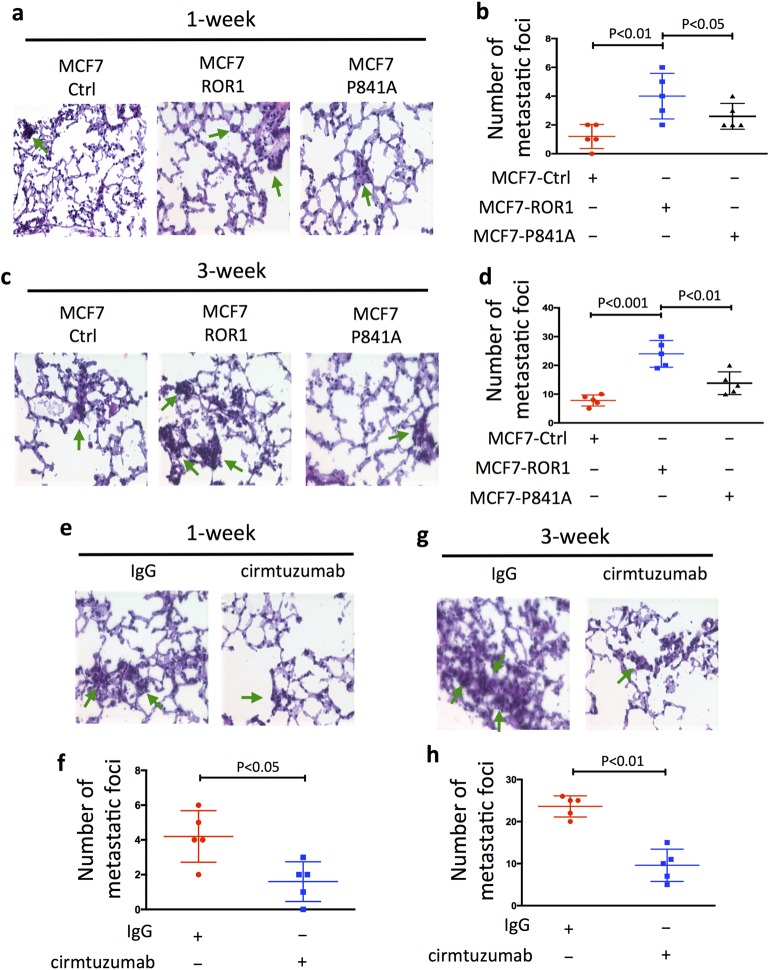
Proline at 841 of ROR1 is critical for enhancing development of metastatic foci of MCF7–ROR1. **a** HE staining of lung tissue from a representative tumor-bearing mouse engrafted with MCF7 or MCF7-expressing ROR1 (MCF7–ROR1) or the mutant form of ROR1, ROR1^P841A^ (MCF7–P841A) at 1 week after intravenous (i.v.) tail-vein injections of equal numbers of viable cells. Green arrows indicate metastatic foci (objective: 40×). **b** Each symbol represents the number of metastatic foci that were found in the lungs of each animal in the groups indicated below (mean ± S.D., *n* = 5). *P* < 0.01; *P* < 0.05, two-tailed Student’s *t* test. **c** HE staining of lung tissue of mice injected as in (**a**), but 3 weeks after i.v. injection of equal numbers of viable cells, as indicated on top. Green arrows indicate metastatic foci (objective: 40×). **d** Each symbol represents the number of metastatic foci found in the lungs of each mouse per group (mean ± S.D., *n* = 5). *P* < 0.001; *P* < 0.01, as assessed by two-tailed Student’s *t* test. **e** HE staining of lung tissue from a representative tumor-bearing mouse injected i.v. with MCF7–ROR1 cells 1 week earlier and treated with nonspecific human IgG (IgG) or cirmtuzumab (10 mg/kg), as indicated on top. Green arrows indicate metastatic foci (objective: 40×). **f** Each symbol represents the number of metastatic foci that were found in the lungs of each animal in each group (mean ± S.D., *n* = 5) (*P* < 0.05, two-tailed Student’s *t* test). **g** HE staining of lung tissue from a representative tumor-bearing mouse injected i.v. with MCF7–ROR1 cells 3 weeks earlier and treated with nonspecific human IgG (IgG) or cirmtuzumab (10 mg/kg), as indicated on top. Green arrows indicate metastatic foci (objective: 40×). **h** Each symbol represents the number of metastatic foci that were found in the lungs of each animal in each group (mean ± S.D., *n* = 5 (*P* < 0.01, two-tailed Student’s *t* test)).

Previous studies demonstrated that ROR1^+^ breast-cancer cells had a greater capacity to metastasize than breast-cancer cells silenced for ROR1.^9^ Consistent with these findings, we found that the numbers of metastatic foci detected in the lungs of mice 1 (Fig. 4a, b) or 3 weeks (Fig. 4c, d) following intravenous injection of MCF7–ROR1 cells were significantly greater than those detected in the lungs of mice injected with equal numbers of MCF7 cells. On the other hand, the numbers of metastatic foci detected in mice injected with MCF7–ROR1^P(841)A^ cells were significantly less than those found in mice injected with MCF7–ROR1 cells (Fig. 4a–d), but comparable to the numbers of metastatic foci detected in mice injected with MCF7 cells, or in mice injected with MCF7–ROR1 cells treated at days-0 and -14 with cirmtuzumab (10 mg/kg) (Fig. 4e–h).

## Discussion

In the present study, we found that Wnt5a induces ROR1 to associate with cortactin, which undergoes tyrosine phosphorylation in breast-cancer PDX cells or MCF7 cells transfected to express ROR1. Moreover, in response to Wnt5a, ROR1/cortactin complexed and stimulated ARHGEF1, which induced activation of RhoA and enhanced cell migration. Silencing expression of cortactin with cortactin-directed siRNA inhibited Wnt5a-enhanced cell migration. Collectively, these studies demonstrate that cortactin plays a critical role in ROR1-dependent, noncanonical Wnt5a signaling, leading to increased tumor-cell migration and metastasis.

The ROR1–PRD was essential for ROR1 to complex with cortactin and increase breast-cancer-cell migration in response to Wnt5a. Cortactin houses a SH3 domain, permitting it to associate with other proteins that have suitable –P–X–X–P– motifs, which typically reside with the PRD.^28–30^ We found that the proline residue at position 841 of ROR1 was indispensable for it to recruit cortactin. Moreover, in contrast to W/T ROR1 or ROR1 with proline-to-alanine substitutions at other sites, the mutant ROR1 with an alanine instead of a proline at position 841 (ROR1^P841A^) was unable to complex with cortactin, enhance cortactin phosphorylation, recruit ARHGEF1, activate RhoA, or enhance cell migration/metastasis of MCF7 cells. Thus, this residue plays an important role for ROR1 to bind and phosphorylate cortactin, which appears necessary to enhance migration and metastasis of MCF7–ROR1 cells. This contrasts with our findings to CLL cells, which also express another cytoskeletal protein named HS1. HS1 also can complex with ROR1 at proline 841 to enhance chemokine-directed migration of CLL cells in response to Wnt5a.^15,16^ HS1 is not expressed in cancers derived from non-hematopoietic cells, such as breast cancer.^17^ Instead, we find that the ROR1–cortactin interaction is critical for enhanced migration and metastasis of breast-cancer cells independent of expression of HS1.

Primary breast cancers that express relatively high levels of phosphorylated cortactin have a greater capacity for migration/metastasis and are associated with a less favorable prognosis than breast cancers with low-to-negligible levels of phosphorylated cortactin.^33–36^ Factors other than Wnt5a, such as activation of Src-family kinases, may also induce cortactin phosphorylation in breast-cancer cells.^39,40^ However, our findings indicate that phosphorylation of cortactin may also be in response to Wnt5a, which is generally present at high levels in breast carcinomas relative to that noted in normal breast tissues.^41,42^ Consistent with this notion, we found that the level of phosphorylated cortactin rapidly attenuated in breast-cancer PDX cultured in serum-free media unless we added exogenous Wnt5a. We find that Wnt5a induces phosphorylation of cortactin at Y421 via ROR1 signaling, which may be dependent on the activity of Src.^37,38^ In any case, our results reveal that the association of cortactin phosphorylation with metastasis may reflect differences in Wnt5a-induced ROR1-dependent signaling. Consistent with this notion are studies demonstrating that high-level expression of ROR1 in breast cancer is associated with increased rates of metastases and poorer survival.^8,9^

We previously found that Wnt5a could induce ROR1 to recruit and activate ARHGEF1, leading to activation of RhoA in leukemia cells.^43^ However, the mechanism for this was unclear, as ARHGEF1 does not contain an SH3-binding domain. Here we demonstrate that association of ARHGEF1 to ROR1 appears mediated by cortactin. In this light, cortactin may be considered an adaptor protein for ROR1, functioning to enhance cell migration and/or metastasis by recruiting and activating ARHGEF1 to ROR1 to provide localized activation of RhoA.^44^ Consistent with this model is the observation that breast-cancer cells with reduced levels of cortactin following treatment with cortactin-specific siRNA had reduced chemotaxis in response to Wnt5a, implicating that cortactin plays a critical role in breast-cancer-cell migration/metastasis.

One study found that Wnt5a could also enhance macrophage-induced invasiveness of MCF7 cells in vitro.^45^ Furthermore, another study found that forced expression of Wnt5a enhanced activation of RhoA in 21PT and 21NT breast-cancer-cell lines, but only increased cellular motility in 21NT cells.^46^ As noted in the present study, MCF7 cells lack expression of ROR1, suggesting that some of the effects of Wnt5a on cell invasiveness may be independent of ROR1. In any case, our study indicates that Wnt5a induces ROR1-dependent cortactin phosphorylation, which prior studies found could associate with the Arp2/3 complex to facilitate cytoskeletal reorganization, stabilized branched actin networks, and lamellipodia formation to enhance cellular motility.^25,47,48^

In conclusion, the present study demonstrates a previously unrecognized ROR1/cortactin/ARHGEF1-dependent pathway leading to activation of RhoA in response to Wnt5a in breast-cancer cells. The reported findings highlight a pathway for potential drug development targeting ROR1-dependent Wnt5a-induced signaling. In this regard, we found that cirmtuzumab could block the capacity of Wnt5a to induce ROR1 to complex and phosphorylate cortactin, recruit ARHGEF1, and activate RhoA, thereby suppressing breast-cancer-cell migration/metastasis. It should be noted that in these studies, cirmtuzumab was injected on the same day on which mice were challenged with cancer cells injected intravenously to study the effect of blocking ROR1 signaling on circulating breast-cancer cells. Other studies have demonstrated that treatment with cirmtuzumab may mitigate the risk for relapse and metastasis of breast-cancer PDX treated with paclitaxel.^49^ Clinical studies are underway to examine the safety and activity of cirmtuzumab used in combination with paclitaxel for treatment of patients with advanced breast cancer (https://clinicaltrials.gov/ct2/show/ NCT02776917). Such studies will be required to determine whether cirmtuzumab may have activity in patients with established metastases. In any case, this study provides added rationale for the clinical evaluation of this humanized anti-ROR1 mAb in the treatment of patients with breast cancer or other ROR1-expressing cancers.^50,51^

## Methods

### Cell culture

MCF7, MDA-MB-231 cells (purchased from ATCC), or MCF7 cells transfected with different ROR1 constructs, were cultured in DMEM medium with 10% FBS, 1% penicillin/streptomycin, maintained at 37 °C in a humidified atmosphere of 5% CO_2_, and tested negative for mycoplasma contamination. Media and supplements were purchased from Life Technologies (Carlsbad, CA, USA).

For serum starvation of breast-cancer PDX cells, freshly isolated PDX cells were cultured in mammary–epithelial basal medium without growth factors, purchased from Lonza.

### Immunoprecipitation analysis

Immunoprecipitation analysis was performed as described.^52^ Cells were lysed in a buffer containing 1% Nonidet P-40, 10 mM Tris-HCl (pH 7.5), 50 mM NaCl, and 1 mM EDTA with protease inhibitors (Roche). The lysates were cleared by centrifugation at 16,000 × *g* for 15 min. Immune precipitates were isolated by using protein A agarose beads, followed by immunoblot or MS analysis. Antibodies for immune precipitation were as follows: the anti-ROR1 antibodies (cirmtuzumab, or 4A5 unless specified) were generated in our laboratory; the anti-cortactin or ARHGEF1 antibody was obtained from Cell Signaling Technology.

### Immunoblot analysis

Immunoblot analysis was performed as described.^52^ Briefly, cells were washed with phosphate-buffered saline (PBS) and suspended in lysis buffer (1% Nonidet P-40, 1 mM EDTA, 50 mM NaCl, and protease inhibitors (Roche) in 10 mM Tris-HCl (pH 7.5)). The cell lysates were cleared by centrifugation at 16,000 × *g* for 15 min and used for immunoblot analysis or for preparing immune precipitates with specific antibodies, which were isolated with protein A agarose. Proteins were separated by sodium dodecyl sulfate polyacrylamide gel electrophoresis and electrophoretically blotted onto polyvinylidene difluoride membrane (Immobilion-P, Millipore). Immunoblot analysis was performed by using primary antibodies specific for cortactin (Cell Signaling Technology, Danvers, MA, USA; dilution 1:1000, catalog#3503), phospho-cortactin (Y421) (Cell Signaling; dilution 1:500, catalog#4569), ARHGEF1 (Cell Signaling; dilution 1:1000, catalog#3669), β-actin (Cell Signaling; dilution 1:1000, catalog#8457), Src (Cell Signaling; dilution 1:1000, catalog#2109), or ROR1 (Cell Signaling; dilution 1:1000, catalog#4102), which were detected with horseradish peroxidase-conjugated secondary antibodies (Cell Signaling). The integrated optical density (IOD) of bands was evaluated by densitometry and analyzed by using Gel-Pro Analyzer software (Media Cybernetics). All the samples for the same experiment were prepared at the same time, and blots were processed in parallel.

### Nucleofection of plasmids and siRNA

Cell line Nucleofector Kit for siRNA or plasmid transfection was purchased from Lonza. Cells (2 × 10^6^) were suspended in 100 μl of Nucleofector Solution with plasmids (pcDNA3.1 vector expressing human ROR1) or siRNAs (GE Dharmacon, Lafayette, CO) and transfected with the Nucleofector II device (program P-020). The transfected cells were cultured in six-well plates in complete medium for 48 (plasmids) or 72 h (siRNA) and then subjected to immunoblot analysis and assays. Endofree Plasmid Maxi Kits (QIAGEN) were used to purify plasmids for transfection. G418 (800 μg/ml) was used for selection of stable MCF7 transfectants, which were then examined via flow cytometry or western blot.

### MS analysis

MS analysis was performed as described previously.^52^ Briefly, bound proteins were digested by trypsin (Roche) directly on beads for the MS analysis. Digested peptides were separated by online 2D-nanoLC and detected by LTQ linear ion-trap mass spectrometers. Each sample took 22.5 h to analyze, and about 200,000 MS/MS spectra were collected for each run. Raw data were extracted and searched by using Spectrum Mill (Agilent, v3.03) database search software against the NCBI ref seq database limited to human taxonomy (version 44).

### RhoGEF nucleotide exchange activity assay

RhoGEF exchange assay kit was purchased from Cytoskeleton and used as per the manufacturer’s instructions. For in vitro guanine nucleotide exchange activity on RhoA, ARHGEF1 was immunoprecipitated from MCF7–ROR1 cells that previously transfected with si-Ctrl or si-Cortactin. Reactions were measured in a Tecan Spectrofluor plus fluorimeter (*λ*_ex_ = 360 nm, *λ*_em_ = 460 nm). Pull-down ARHGEF1 was added after 120 s. Readings were taken at 20 °C every 1 min for a total reaction time of 44 min. The exchange curve can be achieved by exporting raw data to Microsoft Excel and analyzing the data by using GraphPad Prism 6.0.

### RhoA activation assay

RhoA activation assay reagents were purchased from Cytoskeleton and used as per the manufacturer’s instructions. Briefly, GTP-bound active RhoA was pulled down with Rhotekin-RBD beads for 1 h at 4 °C, and then subjected to immunoblot analysis. Immunoblots of whole-cell lysates were used to assess for total RhoA. The IOD of bands was evaluated by densitometry and analyzed by using Gel-Pro Analyzer software (Media Cybernetics).

### Flow cytometry analysis

Flow cytometry analysis was performed as described.^52^ Anti-ROR1 mAb (4A5) conjugated with Alexa Fluor 647 (4A5–Alexa Fluor 647) generated in our laboratory was used to stain cells at 4 °C for 20 min. The stained cells were washed twice with FACS buffer (PBS, pH 7.4, 3% FBS) and examined by 4-color, multiparameter flow cytometry by using a dual-laser FACSCalibur (BD Biosciences). Data were analyzed by using FlowJo software (TreeStar).

### Site-specific mutation

Site-specific mutations were performed as described previously.^53^ In brief, mutation constructs were generated with QuikChange Site-Directed Mutagenesis System (Invitrogen) on the basis of the parental construct (wild-type ROR1), according to the manufacturer’s instructions. The mutations for each construct were verified by DNA sequencing.

The following primer sets were used:

P(784)A, 5′-CAGTGAGTAATCTCAGTAACGCCAGATATC-3′ (sense) and 5′-CATGTAATTAGGATATCTGGCGTTACTGAG-3′ (antisense);

P(808)A, 5′-GATTGCTGGTTTCATTGGCGCGCCAATACC-3′ (sense) and 5′-GGTTCTGAGGTATTGGCGCGCCAATGAAACC-3′ (antisense);

P(826)A, 5′-CAATGGATACCCAATACCTGCTGGATATGCAGC-3′ (sense) and 5′-GGAAACGCTGCATATCCAGCAGGTATTGG-3′ (antisense);

P(841)A, 5-CCAGCCAACAGGTGCTCCCAGAGTGATTC-3 (sense) and 5-GCTGAATCACTCTGGGAGCACCTGTTGG-3 (antisense).

### Cell migration assay

The cell migration assay was performed as described.^13,53,54^ Briefly, cells were collected by treatment with trypsin/EDTA solution, washed twice with serum-free medium, centrifuged, resuspended in medium containing 0.1% bovine serum albumin, and then placed in media at a concentration of 2.5 × 10^4^/mL; each cell suspension was placed onto separate top chambers of a transwell culture polycarbonate insert with 6.5-mm diameter and 8 μm of pore size (Corning). We added Wnt5a at 200 ng/ml in the lower compartment of the chamber. After incubation at 37 °C for 10 h, wells were washed with PBS and fixed with 4% paraformaldehyde. The cells on the apical side of each insert were removed by scraping. Cells that migrated through the pores to the lower chamber were stained with Diff-Quick staining kits (IMEB Inc., San Marcos, CA). Stained cells were analyzed by counting under the Nikon inverted microscope.

### Animal and PDX models

In total, 4- to 6-week-old female Rag2^−/^^−^γ_c_^−/−^ mice were used in this study, following the care and use of laboratory animal guidelines of the National Institutes of Health (NIH). The mice were housed in laminar-flow cabinets under specific pathogen-free conditions and fed ad libitum.

The PDX models were established by using mechanically minced fresh breast-cancer specimens.^55–57^ Early passages of primary-tumor tissues from these PDX models were mechanically minced, and enzymatically and mechanically dissociated by using GentleMACS Dissociator (Miltenyi Biotec) in accordance with the manufacturer’s protocol. Dead cells and erythrocytes were removed through density-gradient centrifugation by using Percoll Plus (CC-17-5442-01; GE Healthcare Life Sciences) following the manufacturer’s protocol.

To test the effects of cirmtuzumab on the tyrosine phosphorylation of cortactin (Y421) in the engraftment of primary breast tumors, 1 × 10^6^ single cells isolated from PDX tumors were suspended in mammary–epithelial growth medium, mixed with Matrigel (BD Biosciences, San Diego, CA) at 1:1 ratio, and injected into the mammary pad of 4- to 6-week-old Rag2^−/−^γ_c_^−/−^ mice. When tumor size reached 200 mm^3^, 10 mg/kg of cirmtuzumab or Ctrl-IgG was injected intravenously biweekly for 1 month. PDX tumors were isolated, lysed, and examined by western blot.

### Metastasis assay

To measure the metastatic potential of MCF7 and various MCF7 transfectants, the cells were injected intravenously into 4- to 6-week-old Rag2^−/−^γ_c_^−/−^ mice treated with estrogen pellets (17β-estradiol, Innovative Research of America, Florida, USA), which were placed subcutaneously in the intrascapular region as described.^58^ MCF7-Ctrl, MCF7–ROR1, or MCF7–P841A cells (1 × 10^6^) were injected intravenously (i.v.) through the lateral tail vein in 100 µl of PBS. At the indicated times (1 or 3 weeks after the injection of cells), the mice were euthanized, and their lungs were removed, fixed, and stained with H&E. Metastatic foci were counted by using Nikon microscope (Japan), objective 40×.

To examine the effect of antibodies on the capacity to generate metastatic foci, MCF7–ROR1 cells (1 × 10^6^) were injected intravenously (i.v.) through the lateral tail vein in 100 µl of PBS. Mice received mg/kg of either Ctrl-IgG or cirmtuzumab on days-0 and -14 at 10 mg/kg via the tail vein (i.v.). At the indicated times (1 or 3 week after the injection of the MCF7–ROR1 cells), the mice were euthanized, and their lungs were removed and fixed in formalin for pathology review, as described in the preceding paragraph.

### Statistical analysis

Data are shown as mean ± S.D. Unpaired two-tailed Student’s *t* test was used to determine the differences between two groups. One-way ANOVA with Bonferroni’s multiple-comparison test was used to calculate the differences between multiple groups of cells. All *P* values < 0.05 were considered significant. GraphPad Prism 6.0 (GraphPad Software Inc.) was used to perform analysis for significance.

### Study approval

Primary breast-tumor specimens were collected from patients, who provided written informed consent on a protocol approved by the Institutional Review Board of the University of California, San Diego (approval number 090401), in accordance with the Declaration of Helsinki. The animal study protocol was approved by the University of California San Diego Institutional Animal Care and Use Committee (approval number S03037).

### Reporting summary

Further information on research design is available in the Nature Research Reporting Summary linked to this article.

## Supplementary information

Supplementary Information

Reporting Summary Checklist

## Data Availability

The data generated and analyzed during this study are described in the following data record: 10.6084/m9.figshare.9874493.^59^ Datasets supporting the figures and tables in this published article are not publicly available to protect patient privacy, but can be accessed from the corresponding author on request, upon the completion of a Data Usage Agreement, as described in the data record above. Raw breast-cancer PDX-derived mass spectrometry data are publicly available in the Japan ProteOme STandard Repository (jPOST repository) under the accession ID: JPST000678. All the uncropped western blots generated during this study are available in Supplementary Fig. 11.
