# Supplementary material for: Wnt5a induces ROR1 to recruit cortactin to promote breast-cancer migration and metastasis
Source: NPJ Breast Cancer. 2019 Oct 25;5:35. doi: 10.1038/s41523-019-0131-9 (PMC6814774; doi:10.1038/s41523-019-0131-9)

SUPPLEMENTARY INFORMATION

Wnt5a Induces ROR1 To Recruit Cortactin To Promote Breast Cancer Migration And Metastasis

Supplementary Figure 1

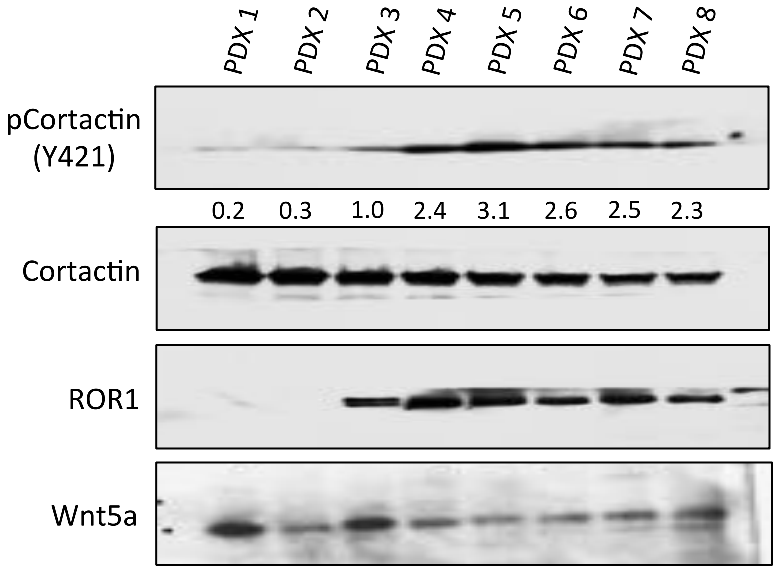

Supplementary Figure 1. Immunoblot analysis of lysates prepared from 8-different breast cancer PDX tumors; the membranes were probed with anti-phospho-cortactin (Y421), anti-cortactin, anti-ROR1, or anti-Wnt5a antibody, as indicated on the left. The numbers between two upper lanes are ratios of band IOD (integrated optical density) of pCortactin (Y421) versus total Cortactin.

Supplementary Figure 2

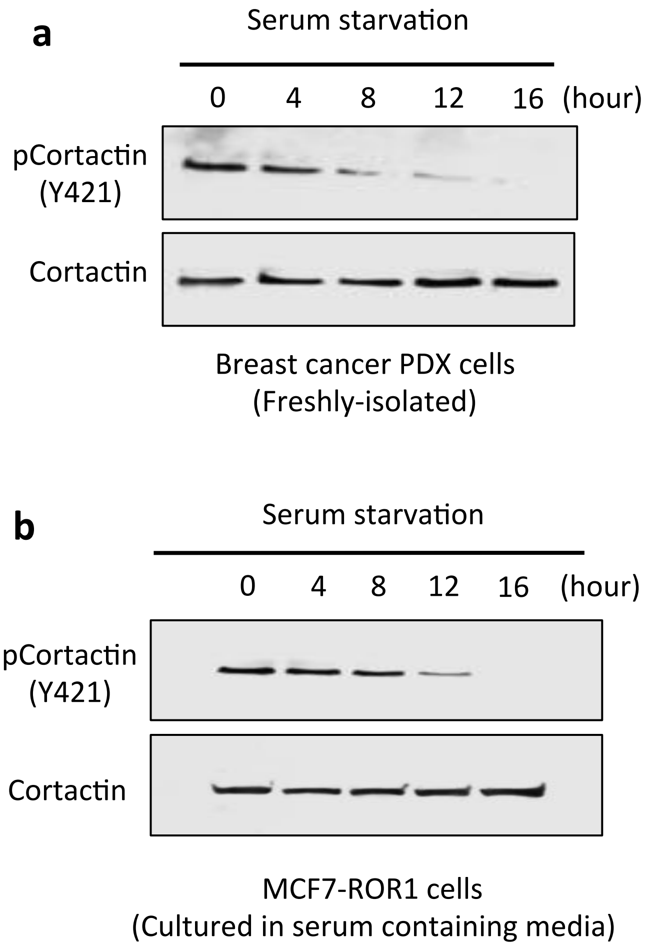

Supplementary Figure 2. a, Immunoblot analysis of lysates prepared from freshly-isolated breast cancer PDX5 cells that were serum-starved for the times indicated on the top (in hours); the membranes were probed with anti-cortactin, or anti-phospho-cortactin (Y421) antibody, as indicated on the left. **b**, Immunoblot analysis of lysates prepared from MCF7-ROR1 cells cultured in 10% serum containing DMEM media that were serum-starved for the times indicated on the top (in hours); the membranes were probed with anti-cortactin, or anti-phospho-cortactin (Y421) antibody, as indicated on the left.

Supplementary Figure 3

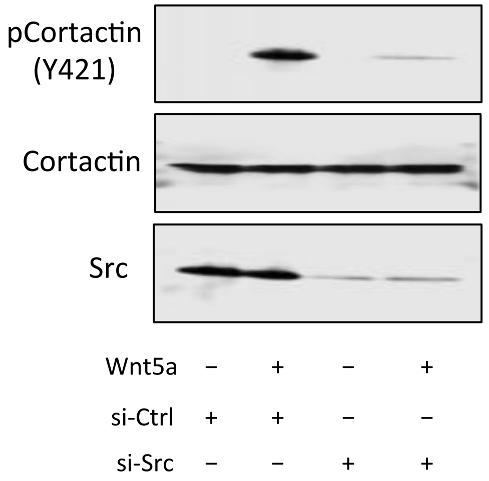

Supplementary Figure 3. Immunoblot analysis of lysates prepared from PDX5 cells transfected 72-hours previously with control siRNA or siRNA targeting Src; that subsequently were treated without (-) or with (+) Wnt5a (100 ng/ml), as indicated at the bottom; the membranes were probed with anti-phospho-cortactin (Y421), anti-cortactin, or anti-Src antibody, as indicated on the left.

Supplementary Figure 4

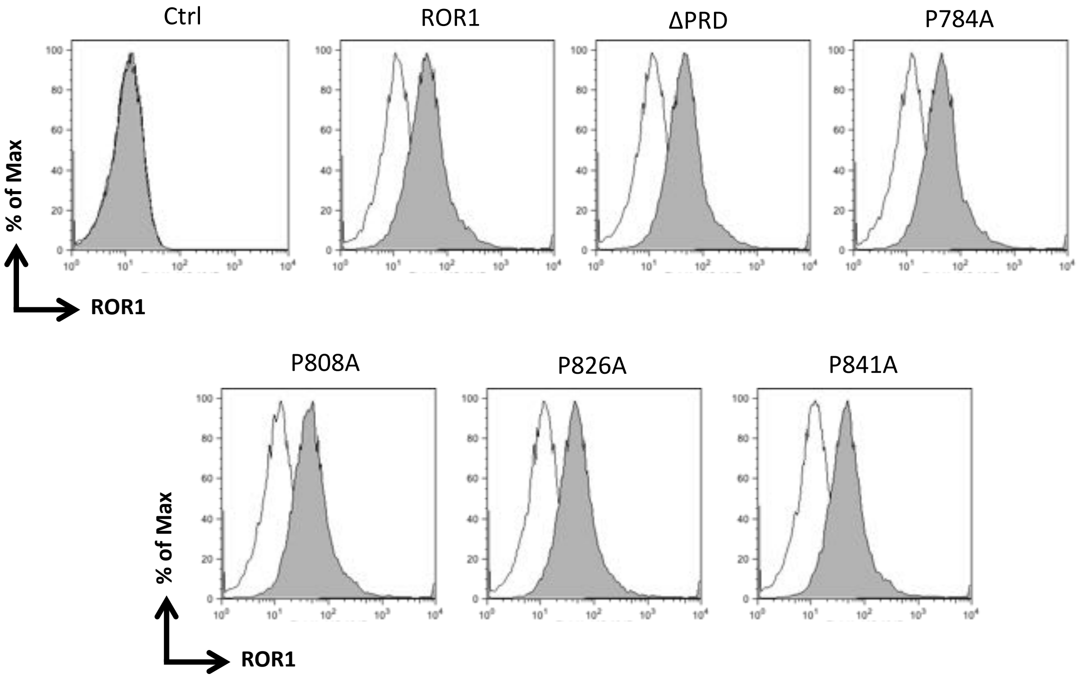

Supplementary Figure 4. Fluorescence of MCF7-Ctrl cells, MCF7-ROR1 (W/T), MCF7-ΔPRD, or MCF7 cells transfected with each of the various mutated forms of ROR1; after staining with a fluorochrome-labeled isotype control mAb (open histograms) or 4A5- Alexa-647 (shaded histograms).

Supplementary Figure 5

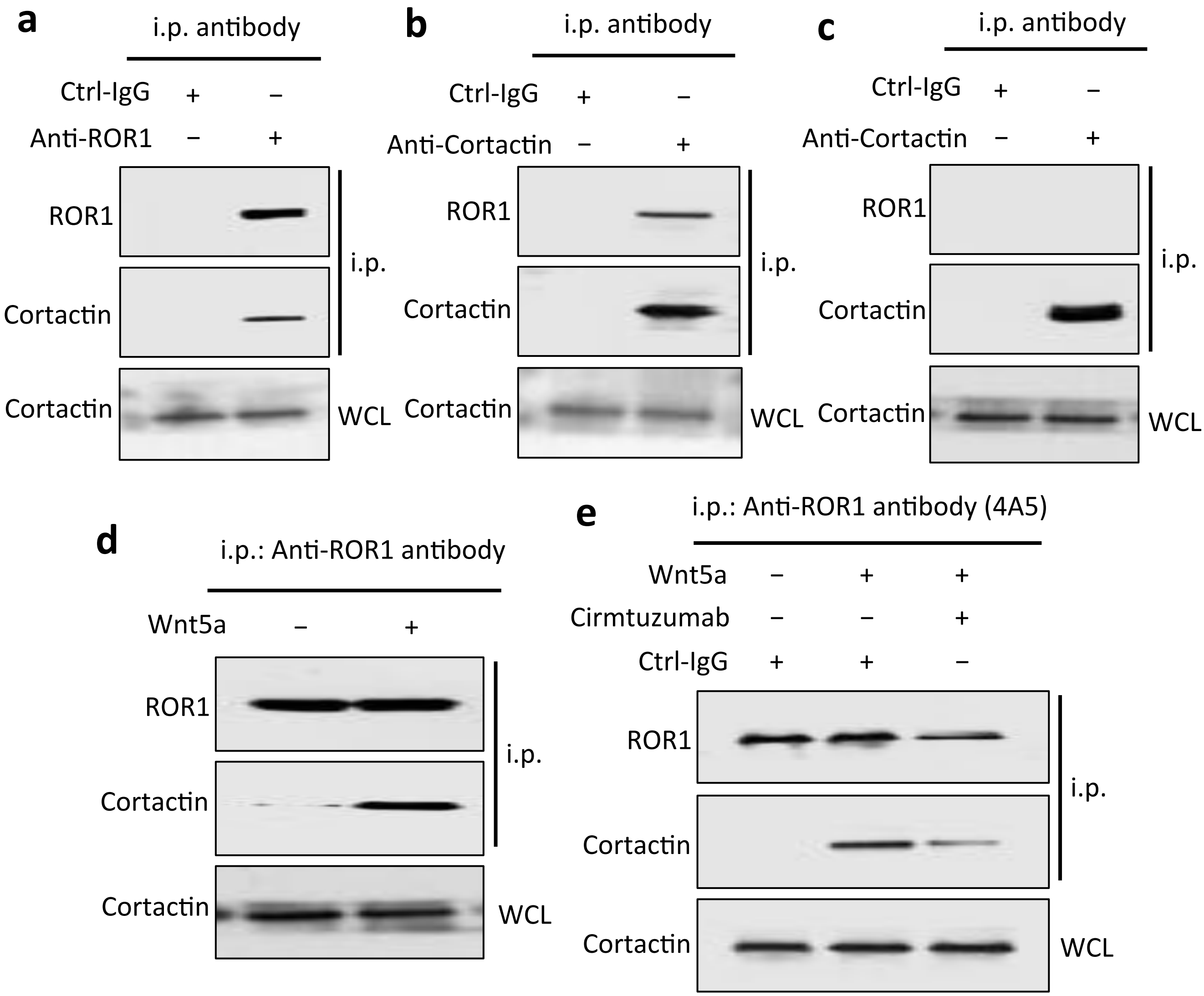

Supplementary Figure 5. Association Of ROR1 With Cortactin In MCF7-ROR1 Cells

a, Immunoblot analysis of anti-ROR1 i.p. or control IgG (Ctrl-IgG) i.p., as indicated at the top, using lysates prepared from MCF7-ROR1 cells; the membranes were probed with anti-ROR1 or anti-cortactin antibody, as indicated on the left. An immunoblot of the whole-cell lysates probed with anti-cortactin mAb is provided in the bottom panel. **b**, Immunoblot analysis of anti-cortactin i.p. or Ctrl-IgG i.p., as indicated at the top, using lysates prepared from MCF7-ROR1 cells; the membranes were probed with anti-ROR1 or anti-cortactin antibody, as indicated on the left. An immunoblot of the whole-cell lysates probed with anti-cortactin mAb is provided in the bottom panel. **c**, Immunoblot analysis of anti-ROR1 i.p. or control IgG (Ctrl-IgG) i.p., as indicated at the top, using lysates prepared from ROR1 negative MCF7 cells; the membranes were probed with anti-ROR1 or anti-cortactin antibody, as indicated on the left. An immunoblot of the whole-cell lysates probed with anti-cortactin mAb is provided in the bottom panel. **d**, Immunoblot analysis of anti-ROR1 i.p. using lysates prepared from overnight, serum-starved MCF7-ROR1 cells that subsequently were treated for 30 minutes without (-) or with (+) Wnt5a (100 ng/ml), as indicated on the top; the membranes were probed with anti-ROR1 or anti-cortactin antibody, as indicated on the left. An immunoblot of the whole-cell lysates probed with anti-cortactin mAb is provided in the bottom panel. **e**, Immunoblot analysis of anti-ROR1 (4A5) i.p. using lysates prepared from overnight, serum-starved MCF7-ROR1 cells that had been treated with Ctrl-IgG or cirmtuzumab (10 µg/ml) for two hours, and subsequently treated for 30 minutes without (-) or with (+) Wnt5a (100 ng/ml), as indicated on the top; the membranes were probed with anti-ROR1 or anti-cortactin antibody, as indicated on the left. An immunoblot of the whole-cell lysates probed with anti-cortactin mAb is provided in the bottom panel.

Supplementary Figure 6

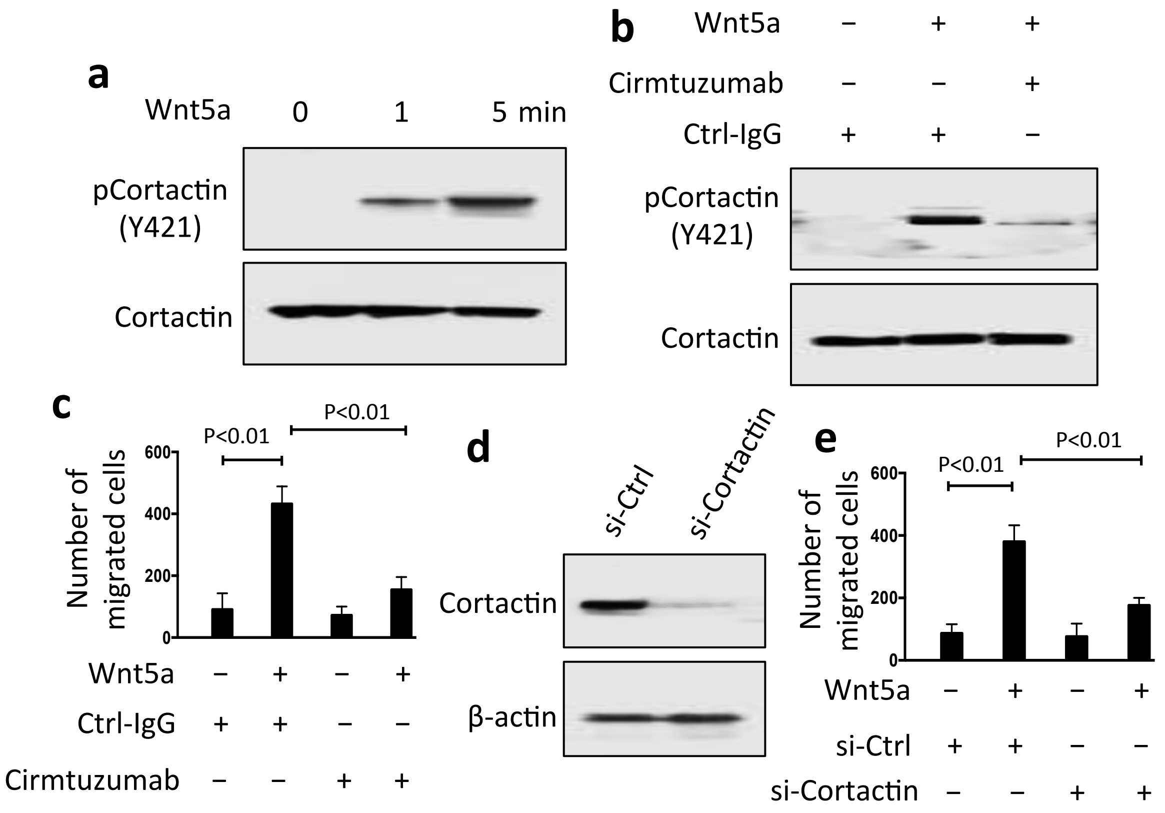

Supplementary Figure 6. Wnt5a Induces ROR1-Dependent Phosphorylation Of Cortactin And Enhances Breast Cancer MCF7 Cell Migration

a, Immunoblot analysis of lysates prepared from overnight, serum-starved MCF7-ROR1 cells that subsequently were treated with Wnt5a (100 ng/ml) for the times indicated on the top (in minutes); the membranes were probed with anti-cortactin or anti-phospho-cortactin (Y421) antibody, as indicated on the left. **b**, Immunoblot analysis of lysates prepared from overnight, serum-starved MCF7-ROR1 cells that subsequently were treated with Ctrl-IgG or cirmtuzumab (10 µg/ml), without (-) or with (+) Wnt5a (100 ng/ml), as indicated on the top; the membranes were probed with anti-cortactin or anti-phospho-cortactin (Y421) antibody, as indicated on the left. **c**, MCF7-ROR1 cells were serum-starved overnight, subsequently treated with Ctrl-IgG or cirmtuzumab (10 µg/ml), and then cell migration assay was performed for 10 hours in the absence (-) or presence (+) exogenous Wnt5a (200 ng/ml), as indicated at the bottom. Data are shown as mean ± S.D. from 3 independent experiments (n=3). $P < 0.01$, as assessed by 2-tailed Student's *t* test. **d**, Immunoblot analysis of lysates prepared from MCF7-ROR1 cells transfected 72-hours previously with control siRNA or siRNA targeting cortactin; membranes were probed with anti-cortactin or anti-β-actin antibody, as indicated on the left. Cell viability was over 85% both in control or cortactin-siRNA transfected cells. **e,** MCF7-ROR1 cell migration was assessed for 10 hours in the absence (–) or presence (+) of exogenous Wnt5a (200 ng/ml), as indicated at the bottom. Data are shown as mean ± S.D. from 3 independent experiments (n=3). $P < 0.01$, as assessed by 2-tailed Student's *t* test.

Supplementary Figure 7

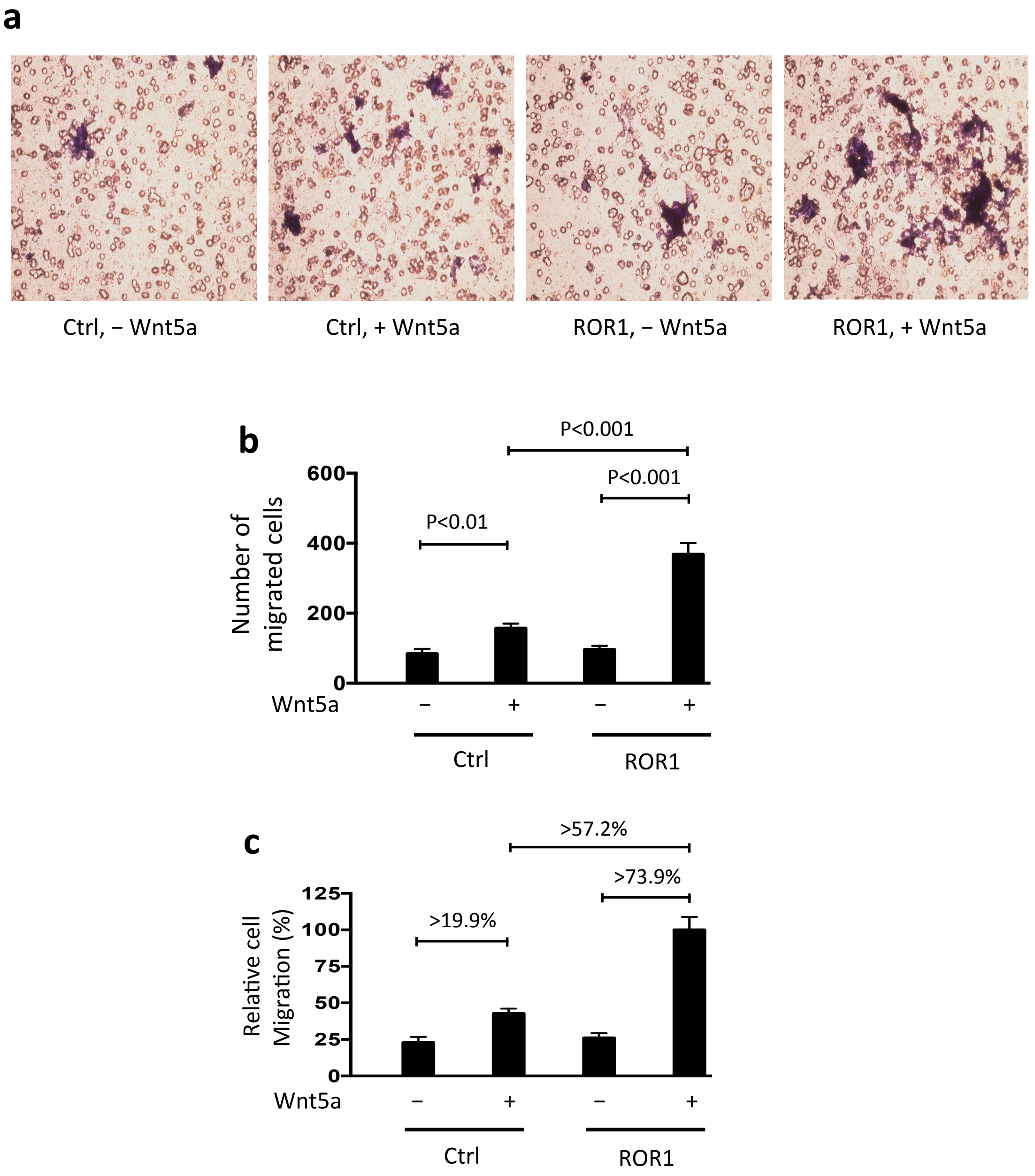

Supplementary Figure 7. a, MCF7-Ctrl or MCF7-ROR1 cells were serum-starved overnight, and cell migration assay was performed for 10 hours in the absence (-) or presence (+) exogenous Wnt5a (200 ng/ml), as indicated at the bottom. Representative photomicrographs of MCF7-Ctrl or MCF7-ROR1 cells in assays for cell-migration. **b**, The histograms depict the relative cell migration as normalized to control. Data are shown as mean ± S.D. from 3 independent experiments (n=3). $P <$ 0.01; $P < 0.001$, as assessed by 2-tailed Student's *t* test. **c**, The histograms represent the normalized percentage (%) of cell migration of panel B.

Supplementary Figure 8

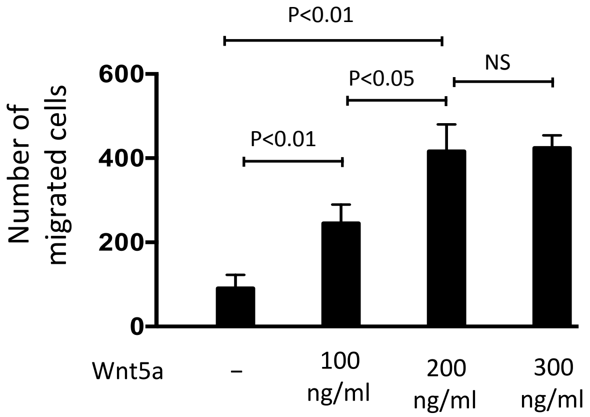

Supplementary Figure 8. MCF7-ROR1 cells were serum-starved overnight, and cell migration assay was performed for 10 hours in the absence (-) or presence (+) of exogenous Wnt5a (100, 200, or 300 ng/ml), as indicated at the bottom. The histograms depict the number of cells migrated. Data are shown as mean ± S.D. from 3 independent experiments (n=3). $P < 0.05$; $P < 0.01$, as assessed by 2-tailed Student's *t* test. 'NS' indicates not significant.

Supplementary Figure 9

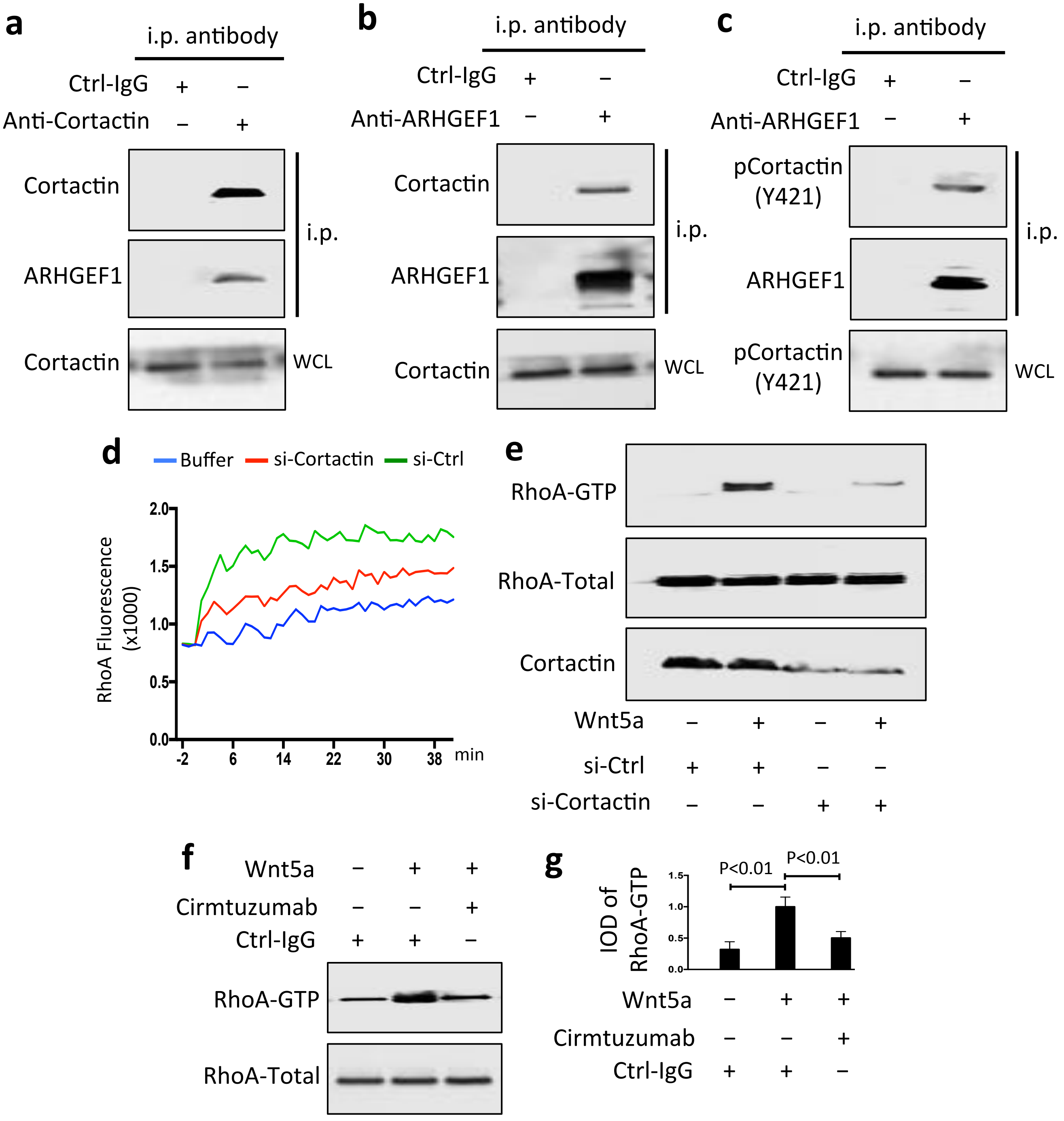

Supplementary Figure 9. Cortactin Associates With ARHGEF1, Which Undergoes Cortactin-dependent Activation To Enhance Activation Of RhoA

a, Immunoblot analysis of anti-cortactin i.p. or Ctrl-IgG i.p., as indicated at the top, using lysates prepared from PDX3 tumor; the membranes were probed with anti-cortactin or anti-ARHGEF1 antibody, as indicated on the left. An immunoblot of the whole-cell lysates probed with anti-cortactin mAb is provided in the bottom panel. **b**, Immunoblot analysis of anti-ARHGEF1 i.p. or Ctrl-IgG i.p., as indicated at the top, using lysates prepared from PDX4 tumor; membranes were probed with anti-cortactin or anti-ARHGEF1 antibody, as indicated on the left. An immunoblot of the whole-cell lysates probed with anti-cortactin mAb is provided in the bottom panel.

c, Immunoblot analysis of anti-ARHGEF1 i.p. or Ctrl-IgG i.p., as indicated at the top, using lysates prepared from PDX5 tumor; membranes were probed with anti-phospho-cortactin (Y421) or anti-ARHGEF1 antibody, as indicated on the left. An immunoblot of the whole-cell lysates probed with anti-phospho-cortactin (Y421) mAb is provided in the bottom panel. **d**, *In vitro* exchange assay on RhoA of anti-ARHGEF1 i.p. from lysates of PDX5 cells transfected with Ctrl-siRNA (green line) or siRNA specific for cortactin (red line) in the presence of Wnt5a. The blue line depicts GTPase-activation using buffer alone. **e**, Immunoblot analysis of lysates prepared from MCF7-ROR1 cells transfected 72-hours previously with control siRNA or siRNA targeting cortactin, that subsequently were treated without (-) or with (+) Wnt5a (100 ng/ml), as indicated at the bottom; expression of cortactin, total RhoA, and activated RhoA was measured, as indicated on the left. **f**, Immunoblot analysis of lysates prepared from overnight, serum-starved breast cancer PDX5 cells (representative of 3 PDXs) that subsequently were treated with Ctrl-IgG or cirmtuzumab (10 µg/ml), without (-) or with (+) Wnt5a (100 ng/ml), as indicated on the top; expression of total RhoA, and activated RhoA was measured, as indicated on the left. **g**, Bars indicate the relative activation of RhoA in breast cancer PDX4, 5, and 6 cells that that had been treated with Ctrl-IgG or cirmtuzumab (10 µg/ml) for two hours, and subsequently treated for 30 minutes without (-) or with (+) Wnt5a (100 ng/ml), as indicated at the bottom. Data are shown as mean ± S.D. of PDX cells from each of 3 different patients. $P < 0.01$, as assessed by 2-tailed Student's *t* test.

Supplementary Figure 10

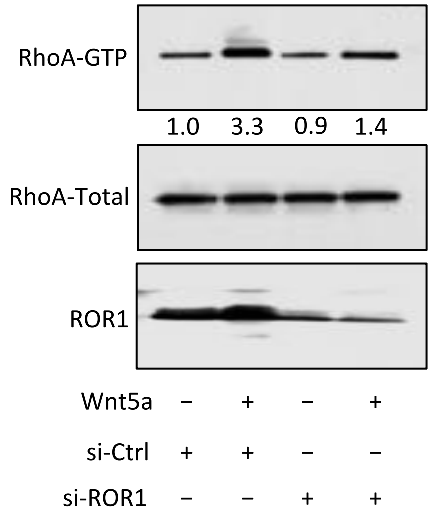

Supplementary Figure 10. Immunoblot analysis of lysates prepared from MDA-MB-231 cells transfected 72-hours previously with control siRNA or siRNA targeting ROR1, that subsequently were treated without (-) or with (+) Wnt5a (100 ng/ml), as indicated at the bottom; expression of ROR1, total RhoA, and activated RhoA was measured, as indicated on the left. The numbers between two lanes are ratios of band IOD (integrated optical density) of GTP-RhoA versus total RhoA.

Supplementary Figure 11: Uncropped blots

Figure 1b

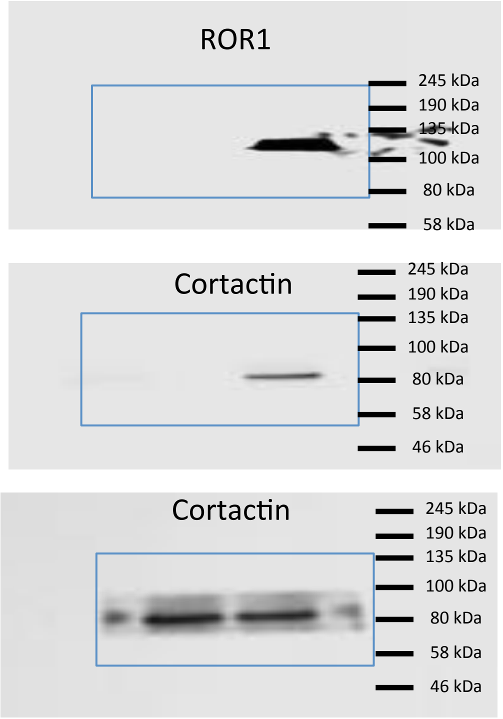

Figure 1d

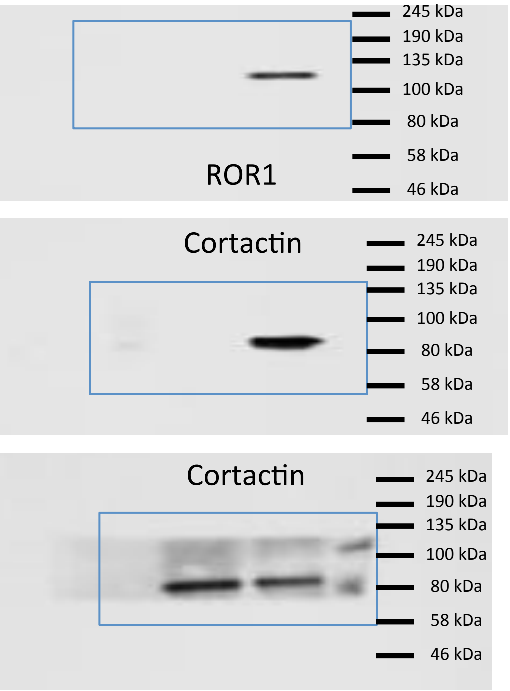

Figure 1f

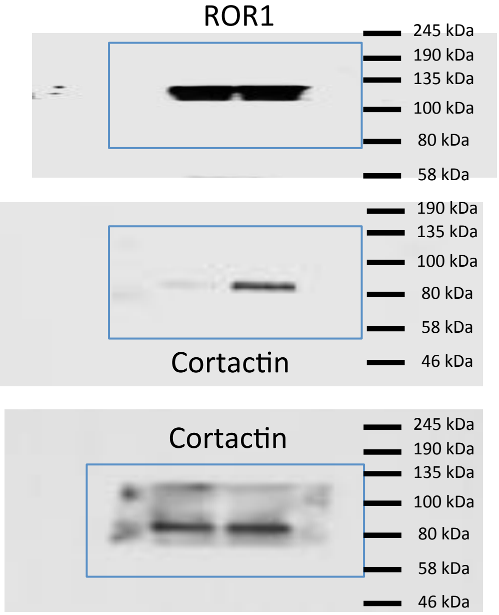

Figure 1h

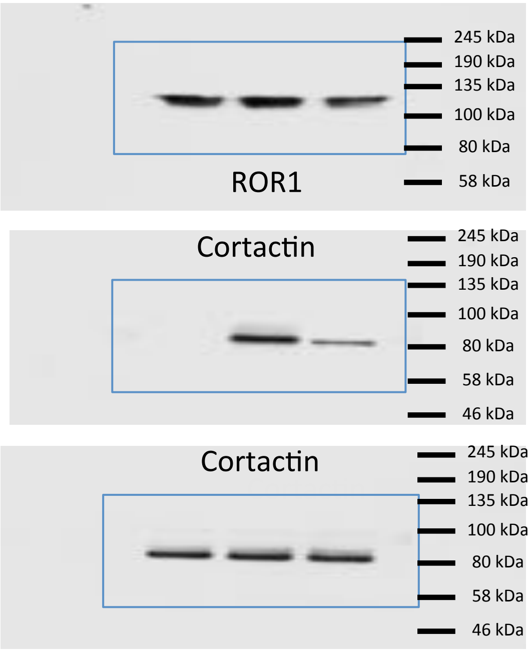

Figure 2a

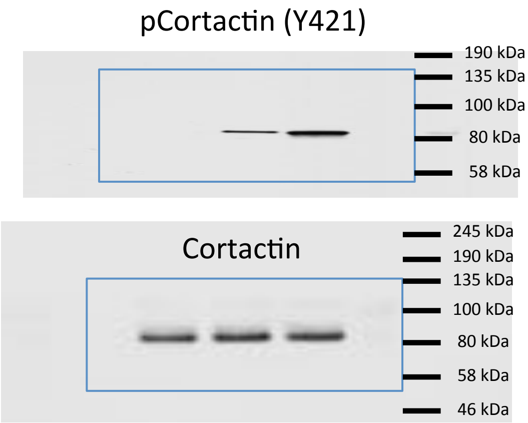

Figure 2c

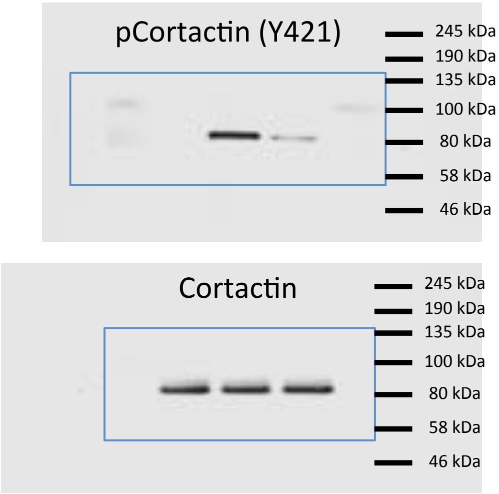

Figure 2e

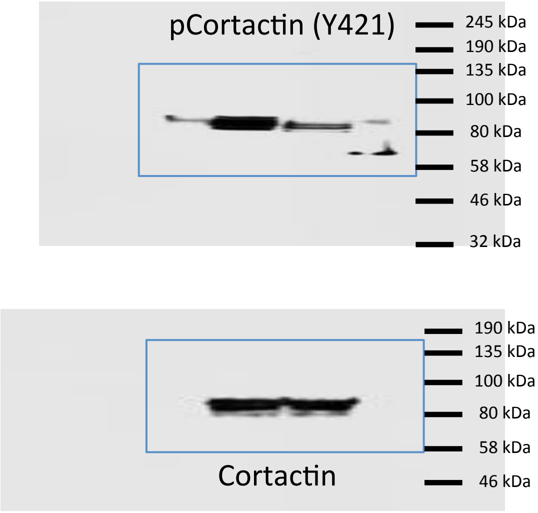

Figure 3d

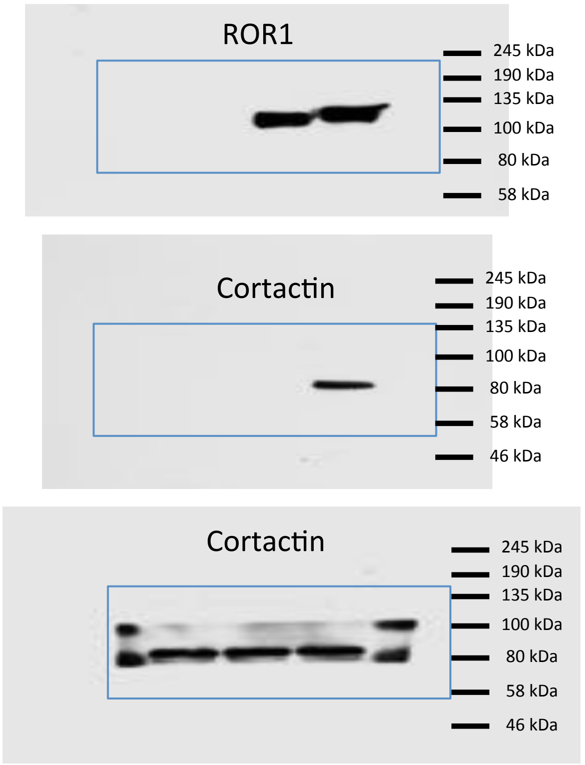

Figure 3e

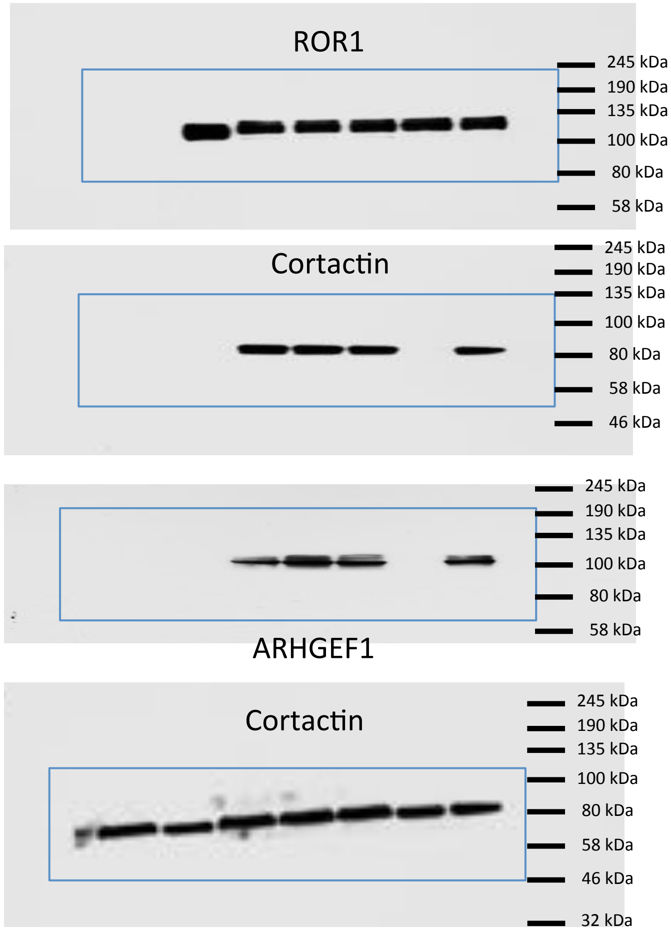

Figure 3f

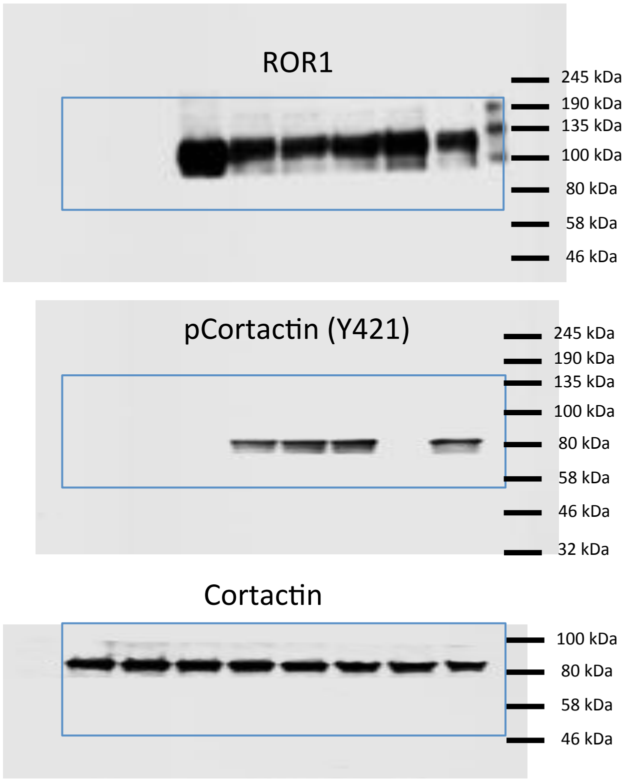

Figure 3g

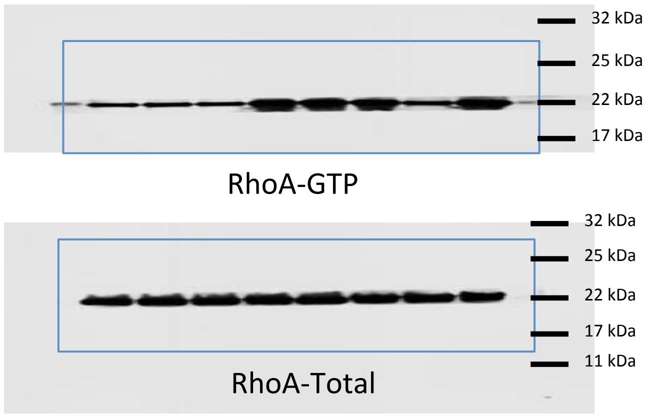

Supplementary Figure 1

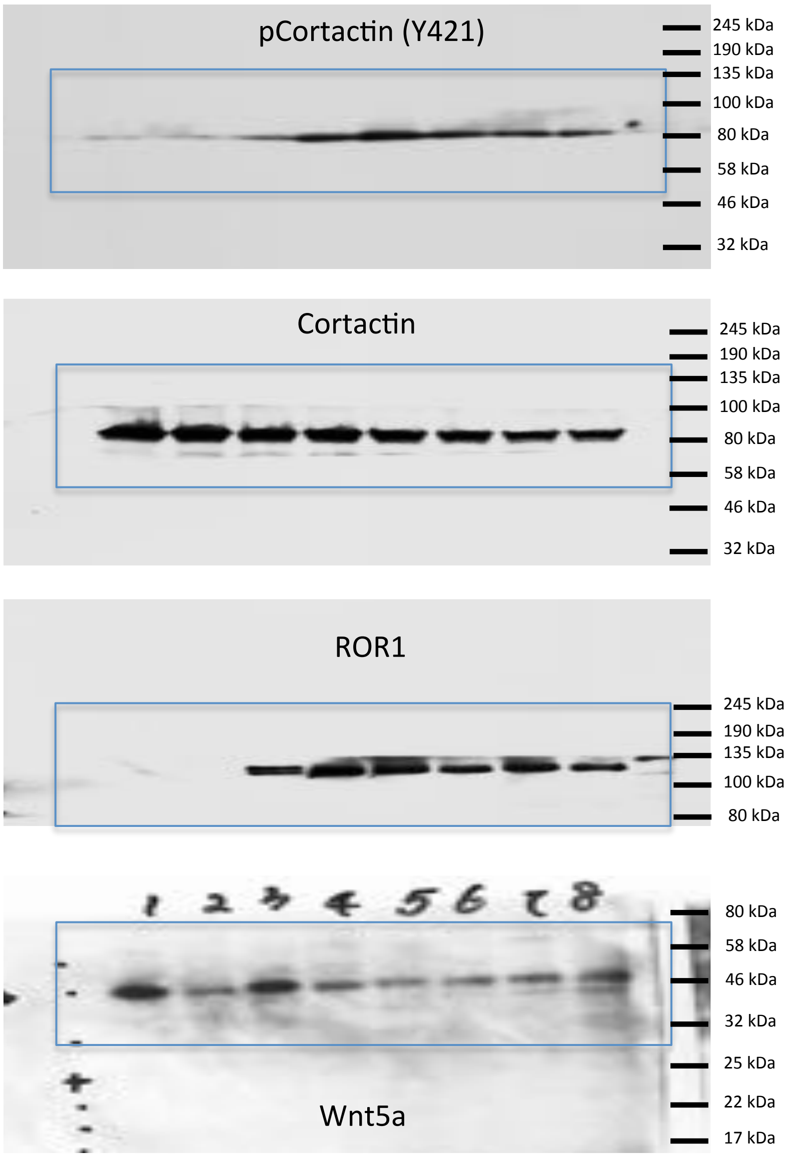

Supplementary Figure 2a

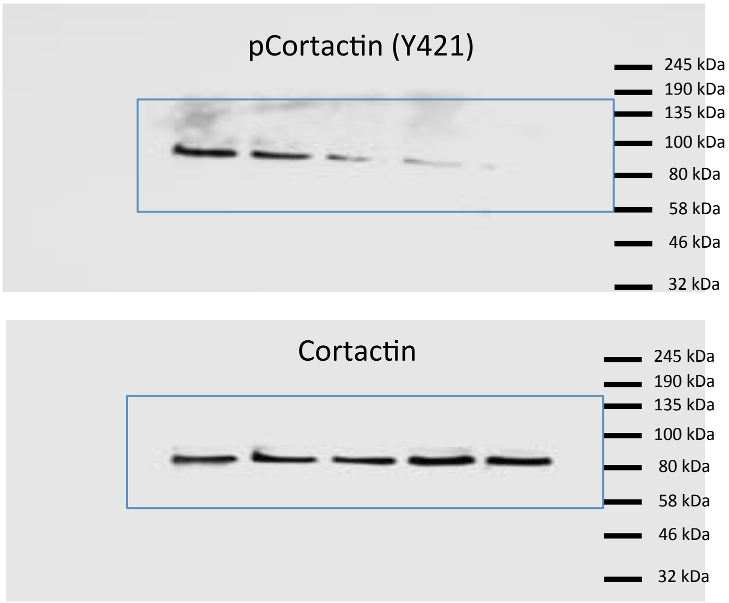

Supplementary Figure S2b

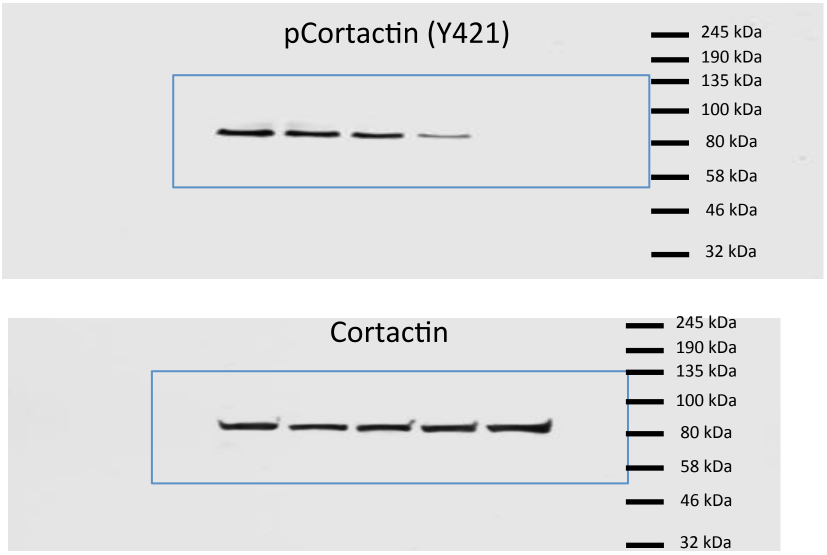

Supplementary Figure 3

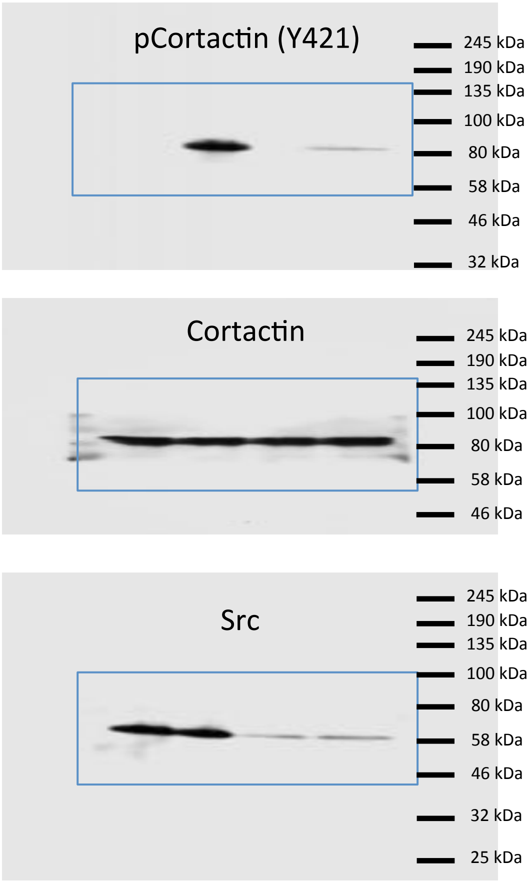

Supplementary Figure 5a

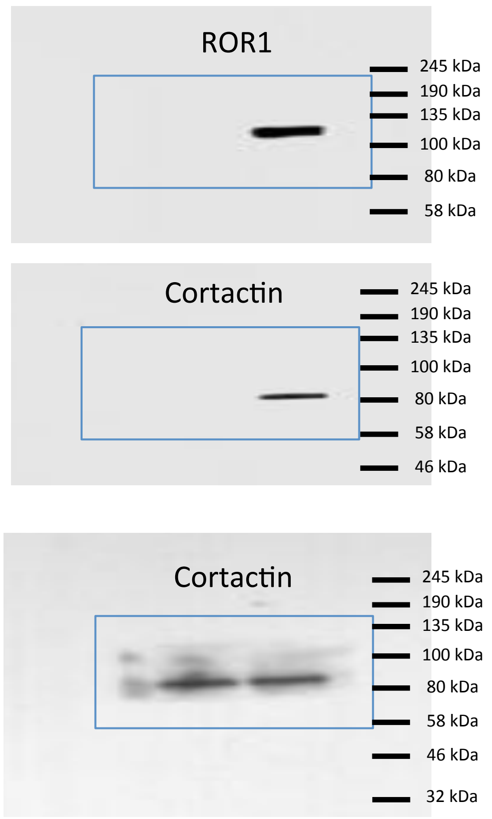

Supplementary Figure 5b

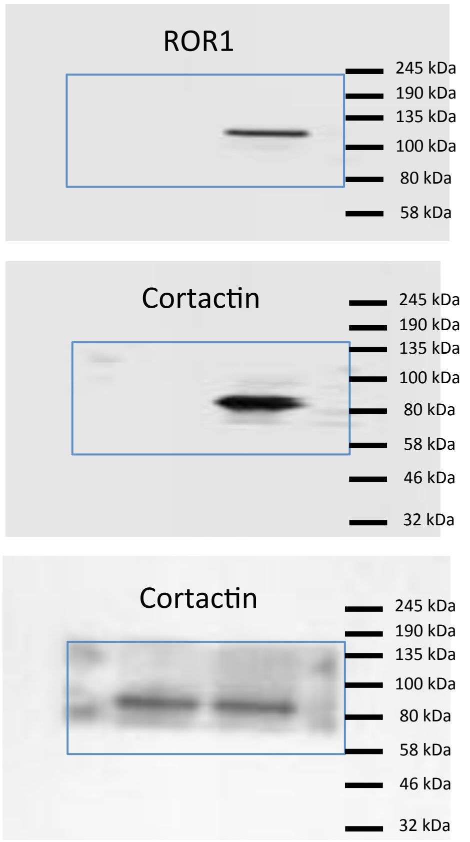

Supplementary Figure 5c

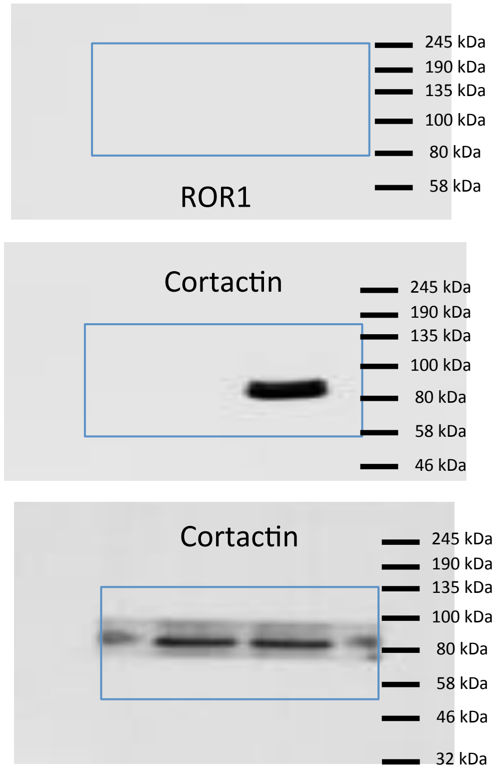

Supplementary Figure 5d

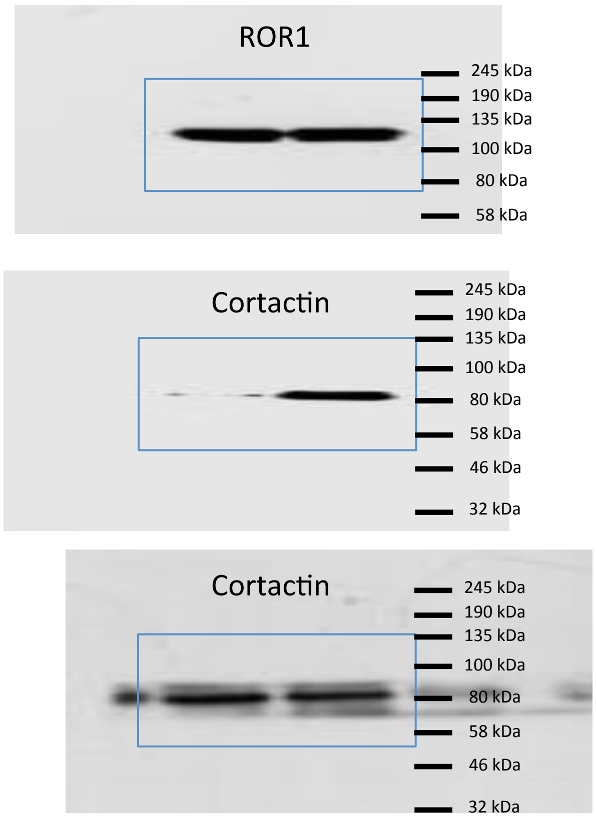

Supplementary Figure 5e

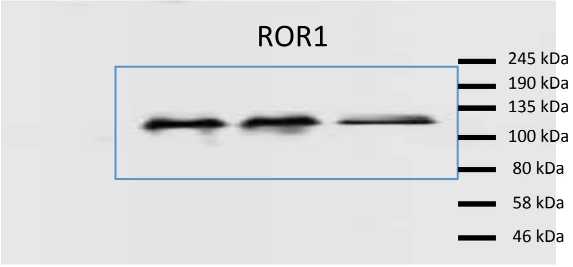

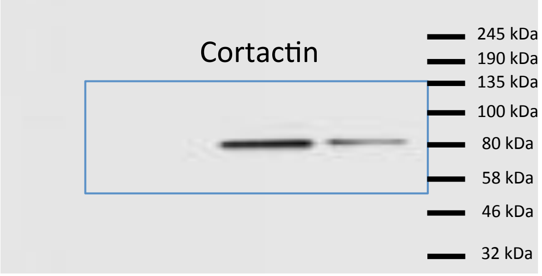

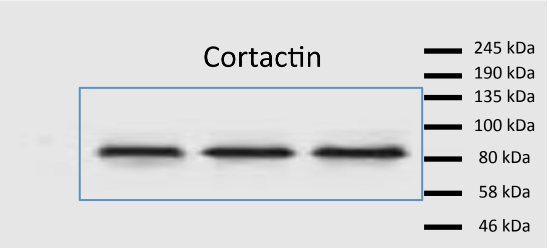

Supplementary Figure 6a

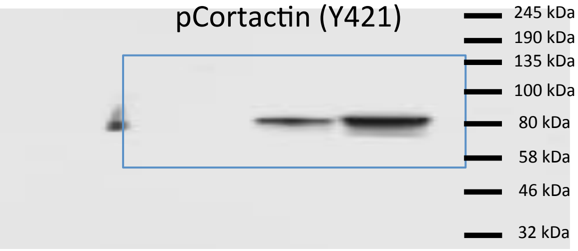

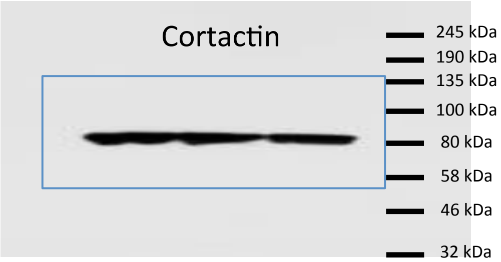

Supplementary Figure 6b

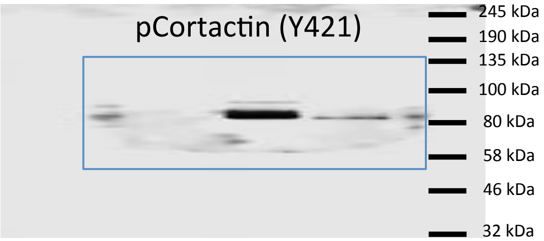

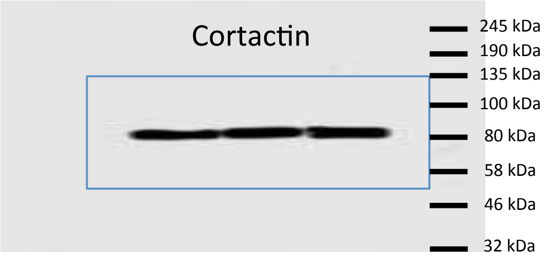

Supplementary Figure 6d

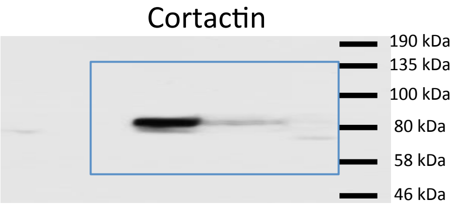

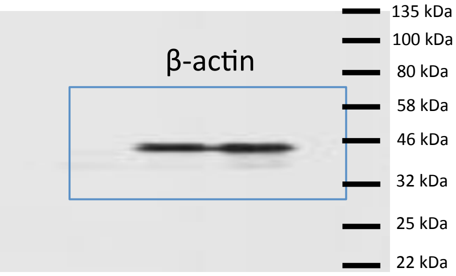

Supplementary Figure 9a

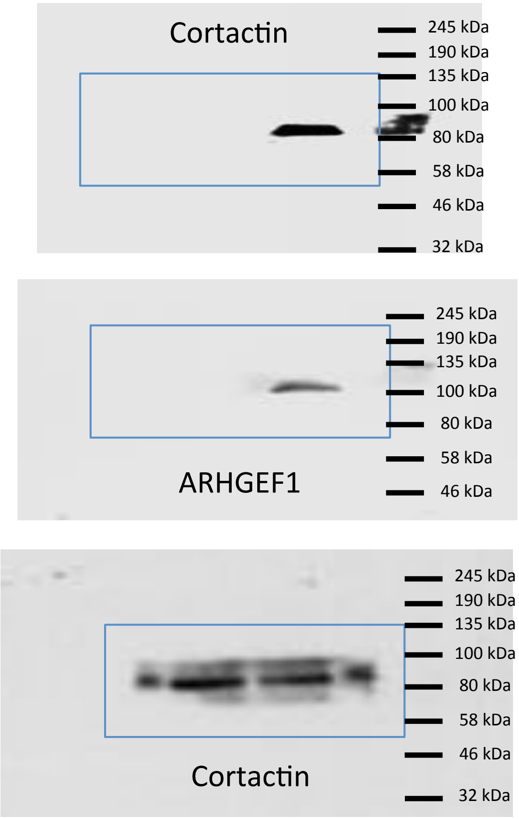

Supplementary Figure 9c

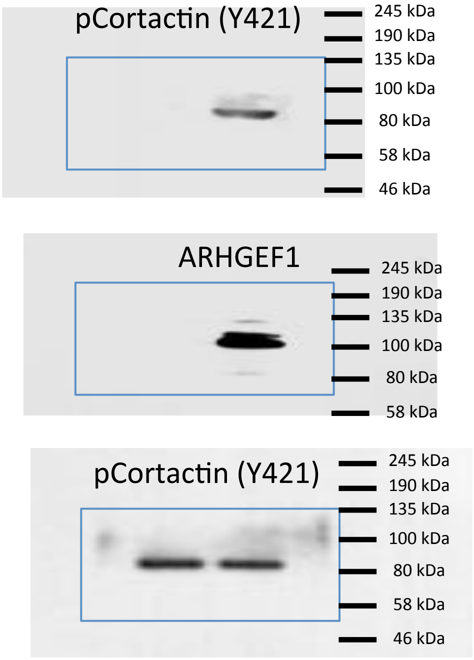

Supplementary Figure 9b

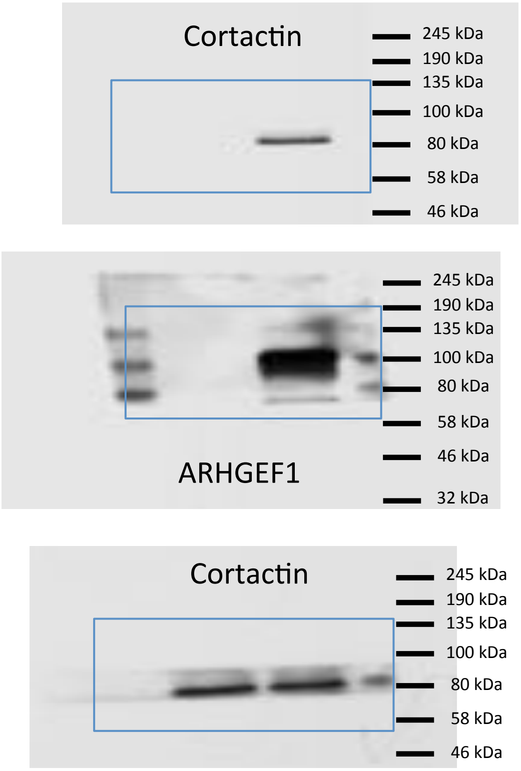

Supplementary Figure 9e

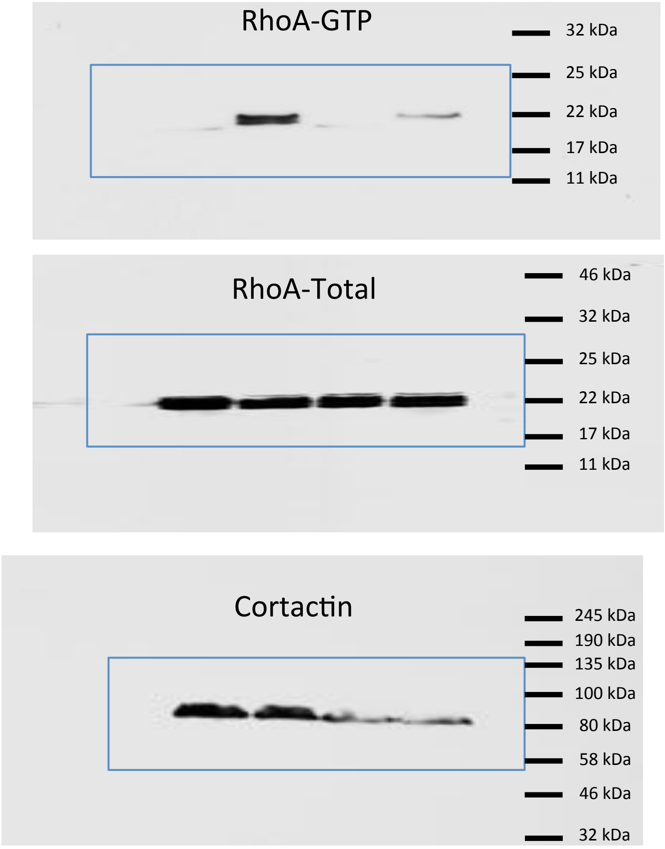

Supplementary Figure 9f

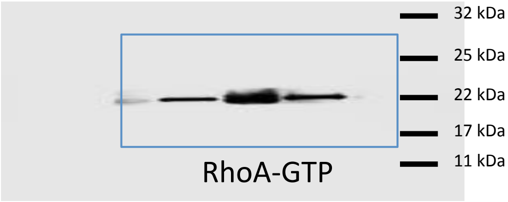

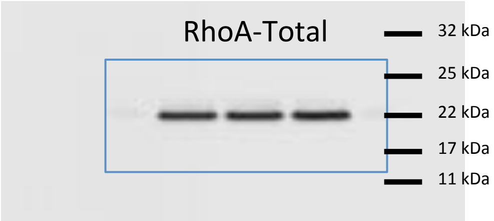

Supplementary Figure 10

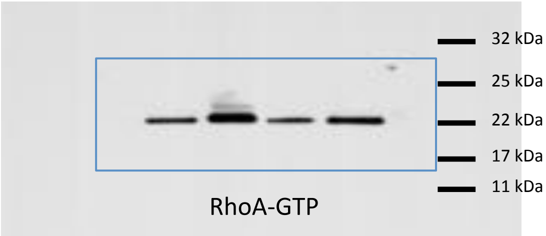

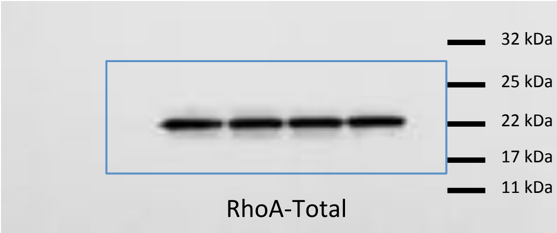

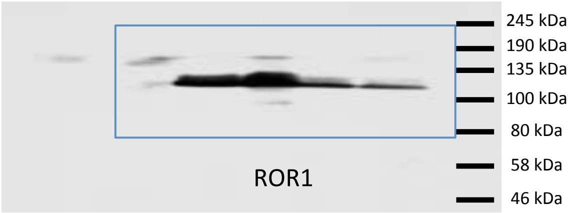

Supplement: Supplementary file 1 — Supplementary Information [file 41523_2019_131_MOESM1_ESM.pdf]
